# NK cells in HPV-related tumorigenesis: mechanisms and clinical applications

**DOI:** 10.3389/fcimb.2025.1723091

**Published:** 2026-01-14

**Authors:** Jianhua Deng, Yu Liu, Xianzong Ma, Daosheng Li, Zhiqi Li, Yuanming Pan, Xiangsheng Zeng

**Affiliations:** 1 Department of Oncology, Jiujiang City Key Laboratory of Cell Therapy, Jiujiang, Jiangxi, China; 2 Senior Department of Gastroenterology, The First Medical Center of Chinese PLA General Hospital, Beijing, China; 3 Department of Gastroenterology, The Seventh Medical Center of Chinese PLA General Hospital, Beijing, China; 4 Cancer Research Center, Beijing Chest Hospital, Capital Medical University/Beijing Tuberculosis and Thoracic Tumor Research Institute, Beijing, China

**Keywords:** HPV-related cancers, immune evasion, natural killer cells, NK cell therapy, tumor microenvironment

## Abstract

Human papillomavirus (HPV) infection is a major global health concern due to its association with various cancers, particularly cervical and head and neck squamous cell carcinomas. High-risk HPV types, such as HPV16 and HPV18, employ oncoproteins E6 and E7 to disrupt host cell regulatory pathways, promote immune evasion, and facilitate malignant transformation. Natural killer (NK) cells, critical components of innate immunity, play a pivotal role in surveilling and eliminating HPV-infected cells. However, HPV-mediated immune evasion mechanisms, including downregulation of MHC-I, suppression of chemokine signaling (e.g., CXCL14), and upregulation of inhibitory molecules (e.g., TIGIT, KLRG1), impair NK cell functionality. This review explores the intricate interactions between HPV and NK cells, highlighting the impact of HPV on NK cell infiltration, exhaustion, and receptor expression. Additionally, it discusses emerging therapeutic strategies to enhance NK cell activation, such as pharmacological agents (e.g., γ-PGA, α-GalCer), innate immune agonists (e.g., STING, RIG-I), genetic engineering (e.g., CAR-NK, iPSC-NK cells), and combination therapies with immune checkpoint inhibitors or monoclonal antibodies (e.g., cetuximab). Clinical applications, including adoptive NK cell transfer and biomarker-guided personalized immunotherapy, are also reviewed. Despite challenges like immunosuppressive tumor microenvironments and limited NK cell persistence, advancements in genetic engineering and nanoparticle delivery systems offer promising solutions. Future research should focus on integrating mechanistic insights with clinical trial design to optimize NK cell-based therapies for HPV-associated malignancies.

## 1 Introduction

### 1.1 Background of HPV infection and its role in tumorigenesis

#### 1.1.1 Epidemiology and high-risk HPV types

Human papillomavirus (HPV) infection represents a significant global health concern due to its high prevalence and association with various cancers, particularly cervical cancer (Soleymaninejadian et al., 2022). Cervical cancer ranks as the second most frequent cancer in women in Mexico, highlighting the regional impact of this disease (Gutiérrez-Hoya et al., 2019). Worldwide, HPV is the causative agent in over 99% of cervical cancer cases (Hus et al., 2015). The global incidence of HPV infections varies geographically, with certain populations and regions being disproportionately affected (Cho et al., 2019). Understanding the epidemiology of HPV infections is crucial for implementing effective prevention and control strategies.

Among the numerous HPV types, high-risk variants, especially HPV16 and HPV18, are predominantly implicated in oncogenesis (Zhang et al., 2019). HPV16 and HPV18 are responsible for approximately 70% of cervical cancer cases globally (Hus et al., 2015). These high-risk HPV types contribute to the development of other anogenital cancers, as well as a subset of head and neck cancers, particularly oropharyngeal squamous cell carcinoma (OPSCC) (Mandal et al., 2016; Wagner et al., 2016). The prevalence of HPV-related OPSCC has been increasing, making it a significant concern in developed countries (Koneva et al., 2018). The distinct biological behavior and improved prognosis of HPV-related OPSCC compared to HPV-negative HNSCC underscore the importance of identifying and understanding the role of HPV in these malignancies (Wagner et al., 2016).

#### 1.1.2 HPV-induced oncogenic mechanisms *via* E6/E7 and immune escape

**Figure 1:**
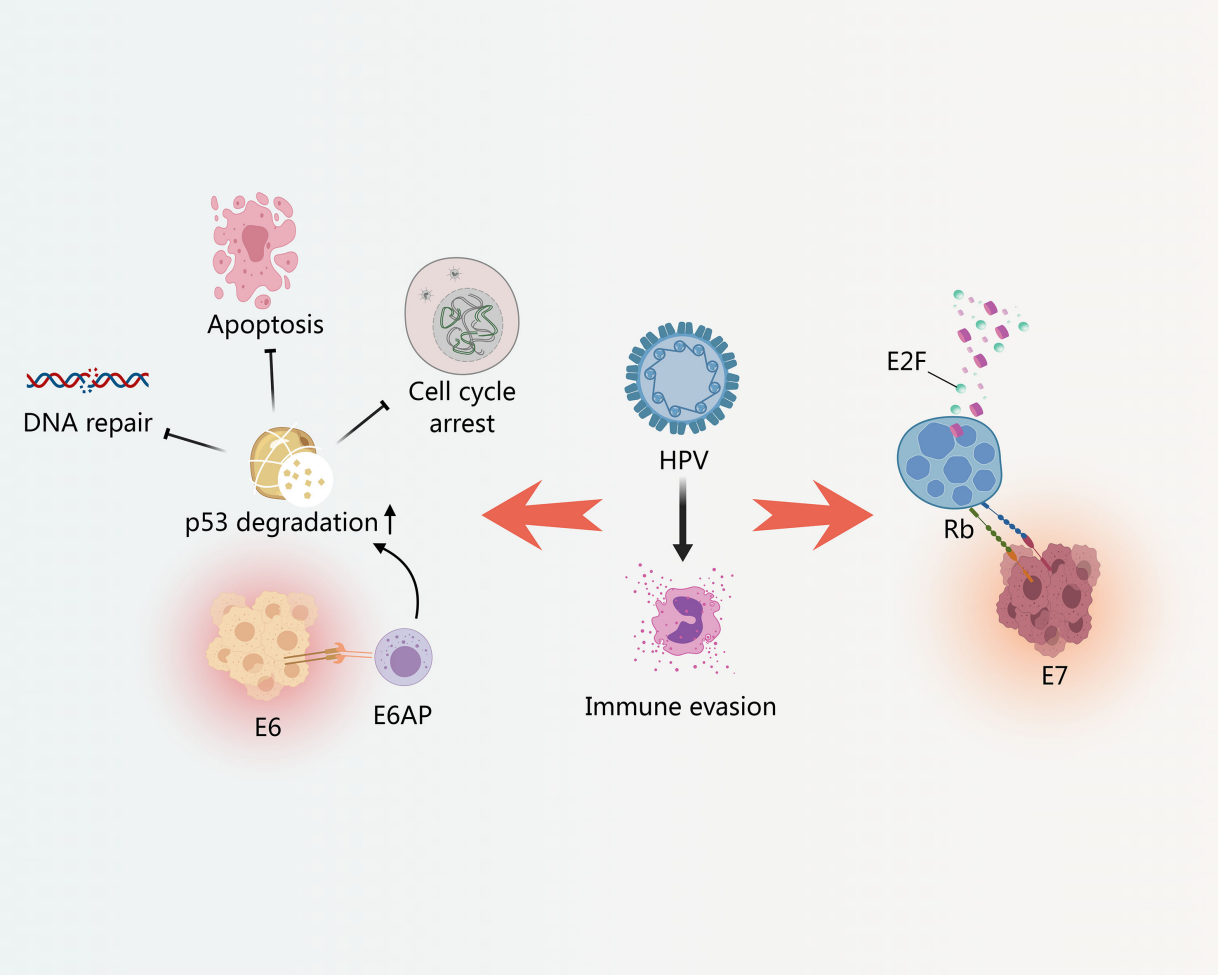
HPV-induced oncogenic mechanisms via E6/E7 and immune escape. E6 promotes the degradation of p53; E7 binds to Rb, releasing E2F transcription factors.

The oncogenic potential of high-risk HPV types is primarily mediated by the E6 and E7 viral oncoproteins (Cicchini et al., 2016). These proteins disrupt crucial host cell regulatory pathways, leading to uncontrolled cell proliferation and genomic instability. Specifically, E6 and E7 interfere with the function of tumor suppressor proteins such as p53 and retinoblastoma protein (Rb), respectively (Cicchini et al., 2016). E6 promotes the degradation of p53, impairing its role in DNA repair, cell cycle arrest, and apoptosis (Cicchini et al., 2016). E7 binds to Rb, releasing E2F transcription factors that drive cell cycle progression, even in the absence of appropriate growth signals (Cicchini et al., 2016) (**Figure 1**).

In addition to their direct effects on cell cycle regulation, E6 and E7 contribute to immune evasion, allowing HPV-infected cells to escape detection and elimination by the host immune system (Westrich et al., 2019). One key mechanism of immune evasion is the downregulation of major histocompatibility complex class I (MHC-I) molecules on the surface of infected cells (Westrich et al., 2019). This reduces the presentation of viral antigens to cytotoxic T lymphocytes (CTLs), impairing T cell-mediated killing of infected cells (Westrich et al., 2019). Furthermore, HPV can modulate the expression of chemokines and cytokines in the tumor microenvironment, suppressing the recruitment and activation of immune cells (Westrich et al., 2019). For example, the expression of CXCL14, a chemokine that recruits natural killer (NK) and T cells, is often downregulated in HPV-positive cancers (Cicchini et al., 2016; Westrich et al., 2019). This downregulation is mediated by the HPV oncoprotein E7 and is associated with DNA hypermethylation in the CXCL14 promoter region (Cicchini et al., 2016).

The impact of these oncogenic and immune evasion mechanisms is significant for the tumor microenvironment. HPV-infected cells can remodel the microenvironment to promote tumor growth and survival (Mukherjee et al., 2018). This includes recruiting immunosuppressive cells, such as myeloid-derived suppressor cells (MDSCs) and regulatory T cells (Tregs), and suppressing the activity of cytotoxic immune cells, such as NK cells and CTLs (Mukherjee et al., 2018; Soleymaninejadian et al., 2022). These changes contribute to a state of chronic inflammation and immune tolerance, facilitating malignant transformation and cancer progression (Zhang et al., 2019).

### 1.2 The role of NK cells in antiviral and antitumor immunity

#### 1.2.1 NK cell cytotoxicity and cytokine secretion

NK cells are a critical component of the innate immune system, playing a vital role in antiviral and antitumor immunity (Soleymaninejadian et al., 2022). NK cells are cytotoxic lymphocytes that can recognize and eliminate infected or transformed cells without prior sensitization (Mandal et al., 2016). This rapid response is essential for controlling early viral infections and preventing tumor development.

NK cells employ several cytotoxic mechanisms to kill target cells. One primary mechanism involves the release of perforin and granzymes. Perforin forms pores in the target cell membrane, allowing granzymes to enter and activate apoptotic pathways. NK cells can also induce apoptosis through the expression of death ligands, such as Fas ligand (FasL) and TNF-related apoptosis-inducing ligand (TRAIL), which bind to death receptors on target cells (Prager and Watzl, 2019). The balance between activating and inhibitory signals received through various receptors determines whether an NK cell will kill a target cell. Activating receptors, such as NKG2D and DNAM-1, recognize stress-induced ligands on target cells, triggering NK cell activation and cytotoxicity. Inhibitory receptors, such as killer cell immunoglobulin-like receptors (molecules on target cells), providing a “self” signal that inhibits NK cell activation (Mariuzza et al., 2025).

#### 1.2.2 The role of CD16 receptor in NK cells

CD16, more accurately known as FcγRIIIA, is a low-affinity IgG Fc receptor (Battella et al., 2016; Bernard et al., 2017; Campbell et al., 2018). It is primarily expressed on NK cells, macrophages, neutrophils, and certain T cell subsets. In NK cells, it is one of the main constitutively expressed activating receptors. It is a transmembrane protein that non-covalently associates with two homologous or heterologous signaling subunits (most commonly CD3ζ and FcRγ). The intracellular regions of these subunits contain immune receptor tyrosine-based activation motifs (ITAMs), which are essential for initiating downstream signaling (Rostamzadeh et al., 2018; Chong and MacKerell, 2022; Aguilar et al., 2024).

#### 1.2.3 The core mechanism of antibody dependent cell cytotoxicity

ADCC serves as an important bridge between adaptive immunity (antibodies) and innate immunity (NK cells) (de Taeye et al., 2020; Li and Liu, 2022; Khoshtinat Nikkhoi et al., 2024). The process can be summarized in the following steps:

1. Antibody Binding: When pathogens infect or cells undergo malignant transformation, the body produces specific IgG antibodies. These antibodies bind to specific antigens on the surface of target cells through their Fab region (Sun et al., 2021).
2. Fc Region Exposure: After binding, the Fc region of the IgG antibody undergoes a conformational change and becomes exposed, forming a “flag” (Dixon et al., 2021).
3. CD16 Recognition and Cross-linking: The CD16 receptors on the surface of NK cells recognize and bind to these exposed IgG Fc regions. When multiple CD16 molecules on an NK cell surface bind to several adjacent IgG Fc regions simultaneously, receptor cross-linking occurs, which is a key trigger for activation signaling (Kamen et al., 2022).
4. Signal Transduction:
  - Cross-linking leads to the phosphorylation of the CD16-associated ITAM motifs (on CD3ζ and FcRγ subunits) by Src family kinases (such as Lck) (Aguilar et al., 2022).
  The phosphorylated ITAM recruits and activates Syk family tyrosine kinases (Syk and ZAP-70) (Millan et al., 2025).
5. Activation of Signaling Pathways:
  PLC-γ Pathway: Activated Syk/ZAP-70 phosphorylates and activates phospholipase C-γ (PLC-γ). PLC-γ hydrolyzes phosphatidylinositol 4,5-bisphosphate (PIP2) to produce inositol trisphosphate (IP3) and diacylglycerol (DAG) (Ting et al., 1995; Caraux et al., 2006; Pugh et al., 2018).
  · IP3 prompts the endoplasmic reticulum to release calcium ions (Ca^2+^), leading to a sharp increase in intracellular calcium concentration (Lin et al., 2019).
  · DAG and calcium ions together activate protein kinase C (PKC) (Hsu et al., 2023).
  · MAPK Pathway: Simultaneously, downstream signaling also activates the Ras-MAPK (such as ERK) pathway (Shokouhifar et al., 2021; Watkinson et al., 2021; Bahar et al., 2023).
  · PI3K Pathway: The phosphorylated ITAM can also recruit and activate phosphatidylinositol 3-kinase (PI3K), producing PIP3, which in turn activates signaling molecules such as Akt (Gallardo-Vera et al., 2018).

#### 1.2.4 Regulation of NK cell cytotoxic activity

The activation of the aforementioned signaling pathways directly leads to the outbreak of NK cell cytotoxic effects, mainly through two methods:

1. Release of perforin and granzymes (Shevtsov and Multhoff, 2016; Prager and Watzl, 2019; Ijaz et al., 2023; Chen et al., 2024):
  · Polarization: Cytoskeletal reorganization leads to the movement and polarization of cytotoxic granules toward the immune synapse in contact with the target cell.
  · Exocytosis: A sharp rise in calcium ions is a direct signal triggering the fusion of cytotoxic granules (containing perforin and granzymes) with the cell membrane and their release into the synaptic cleft.
  · Function: Perforin forms pores on the target cell membrane, allowing granzymes (a series of serine proteases) to enter the target cell, inducing apoptosis through various pathways, such as the activation of the Caspase cascade.
  · This is the primary and fastest mechanism for ADCC-mediated killing of target cells.
2. Upregulation of death receptor pathways (García-Lora et al., 2003; Li et al., 2019; Imaoka et al., 2022; De Federicis et al., 2024; He et al., 2024):
  · Activation signals (especially PKC and MAPK pathways) can also upregulate the expression of FasL and TRAIL on NK cell surfaces.
  · These ligands bind to the corresponding death receptors (Fas, DR4/5) on the target cell, which can also initiate the apoptosis program of the target cell. This pathway serves as a complementary mechanism for cytotoxicity.

#### 1.2.5 Regulation of cytokine and chemokine secretion

The activation of CD16 is not only a signal for “killing,” but also a signal for “crying for help” and “coordination”. It strongly induces NK cells to secrete various cytokines and chemokines:

Key factors:

IFN-γ: the most important product. It is mainly induced by the collaboration of MAPK (ERK) and PI3K pathways. IFN-γ strongly activates macrophages, enhances antigen presentation, and shapes Th1 immune responses, which are crucial in antiviral and antitumor immunity (Capuano et al., 2020). TNF-α: induced by similar pathways, it can directly kill certain target cells and promote local inflammatory responses (Mujal et al., 2025). GM-CSF: granulocyte-macrophage colony-stimulating factor, promotes the development and activation of myeloid cells (such as dendritic cells) (Lian et al., 2024). Chemokines such as CCL3/MIP-1α and CCL4/MIP-1β can recruit other immune cells (such as monocytes and T cells) to sites of inflammation or tumors, amplifying the immune response (Sun et al., 2024; Ladd et al., 2025).
Regulatory mechanisms: The transcriptional activation of cytokine genes relies on the integration of multiple signaling pathways. For example, the production of IFN-γ requires the combined input of PKC, ERK, and calcium signals (Lee et al., 2022; Picard et al., 2022; Khanal et al., 2025).

CD16-mediated ADCC is one of the core functions of NK cells, transforming the specificity recognized by antibodies into powerful effector functions through a sophisticated signaling network (Karampatzakis et al., 2021; Liu, 2022; Cocker et al., 2023; Nikkhoi et al., 2023):

Cytotoxicity: It activates calcium influx and PKC via the PLC-γ/IP3/DAG pathway, driving the release of perforin/granzyme and aiding the death receptor pathway for efficient and specific killing of target cells.
Cytokine secretion: It induces the production of large amounts of IFN-γ, TNF-α, etc., through pathways like MAPK and PI3K, thereby regulating and amplifying the overall adaptive immune response.

#### 1.2.6 The unique role and significance of IL-18 and IL-21 in the amplification of NK cells

In-depth exploration of the unique roles and significance of IL-18 and IL-21 in the proliferation of NK cells (**Table 1**). As mentioned earlier, the activation of CD16 primarily drives the immediate effector functions of NK cells (ADCC killing and cytokine secretion). However, to maintain a lasting immune response, the proliferation, survival, and long-term functional maintenance of NK cells are crucial. At this point, cytokines such as IL-18 and IL-21 play an indispensable role. They synergize with the CD16 signaling to collectively determine the scale, quality, and duration of the NK cell response (Anderko et al., 2020; Harper et al., 2021; Liu et al., 2021; Sugawara et al., 2023; Tang et al., 2023).

**Table 1:** Summary and comparison of IL-18 and IL-21.

Characteristics	IL-18 (Anderko et al., 2020; Anderko et al., 2020; Liu et al., 2021; Sugawara et al., 2023; Cheng et al., 2024)	IL-21 (Harper et al., 2021; Tang et al., 2023)
Main Source	Macrophages, dendritic cells	Activated CD4+ T cells (Tfh, Th17)
Core Role	A powerful co-stimulatory factor driving inflammatory responses and memory-like differentiation	A fine-tuning regulatory factor that collaborates to promote maturation and expansion, but limits long-term survival
Relationship with CD16	Enhances IFN-γ production and proliferation responses triggered by CD16	Can be secreted by activated T cells, acting in a “paracrine” manner on NK cells to optimize their ADCC function
Role in Amplification	In combination with IL-12/15, produces long-lasting, memory-like NK cells suitable for adoptive therapy	In combination with IL-15, used for short-term, high-efficiency *in vitro* amplification to obtain a large number of highly cytotoxic NK cells
Key Signals	MyD88/NF-κB/MAPK	JAK/STAT (STAT1, STAT3)

**Core concept: From “activation” to “amplification”**

CD16 signal: Provides a “trigger signal” for specific activation and effector function.
IL-18/IL-21 signal: Provides co-stimulatory signals that drive clonal expansion, functional maturation, and survival, equivalent to “accelerator” and “maintenance signal.” (**Table 1**)

**The significance of IL-18 in the expansion of NK cells**

IL-18 belongs to the IL-1 cytokine family and is a potent pro-inflammatory cytokine primarily produced by activated macrophages and dendritic cells.

1. As a powerful co-stimulatory factor:
  Synergistic action: IL-18 has a relatively weak capacity to promote the proliferation of resting NK cells on its own, but it can greatly synergize with IL-12, IL-15, or CD16 signals to strongly enhance NK cell proliferation and IFN-γ production (Anderko et al., 2020; Cheng et al., 2024).
  Mechanism: After binding to its receptor (IL-18R), IL-18 activates the NF-κB and MAPK pathways through the MyD88/IRAK/TRAF6 pathway. When combined with signals from IL-12 (activating STAT4) or IL-15 (activating STAT5), it produces a “supra-additive effect”, maximizing the transcription and synthesis of IFN-γ while strongly driving the cell cycle process (Khan et al., 2021; Terrén et al., 2023).
2. Promoting NK cell survival and metabolic reprogramming
  IL-18 signaling can upregulate the expression of anti-apoptotic proteins (such as Bcl-2 and Bcl-xL), thereby extending the lifespan of NK cells and creating conditions for multiple rounds of proliferation. It can also promote the metabolic shift of NK cells from oxidative phosphorylation to aerobic glycolysis, a “Warburg effect” that provides necessary biosynthetic precursors for rapidly proliferating cells (Berjis et al., 2024; Casagrande Raffi et al., 2024; Shen et al., 2025).
3. Role in the generation of “memory-like” NK cells
  This is a very crucial role of IL-18. When NK cells are pre-activated by a combination of IL-12, IL-15, and IL-18 cytokines, they differentiate into a type known as “cytokine-induced memory-like NK cells.” These memory-like NK cells survive long-term in the body and exhibit a more robust and rapid proliferative capacity and effector function upon re-encountering activation signals (such as through CD16 or cytokine receptors), resembling the “memory” characteristics of adaptive immunity. This has great application value in adoptive immunotherapy (Tarannum and Romee, 2021; Cubitt et al., 2022; Terrén et al., 2022; Foltz et al., 2024).

**The significance of IL-21 in NK cell expansion**

IL-21 is primarily produced by activated CD4+ T follicular helper cells (Tfh) and Th17 cells, and which is an important cytokine for regulating humoral and cellular immunity. Its effects on NK cells are quite unique, exhibiting a “double-edged sword” effect that highly depends on the contextual environment (Heinze et al., 2019; Ma et al., 2022; Maia et al., 2024; Tran et al., 2025).

1. Synergistically drives terminal maturation and early expansion with IL-15
  Strongest synergistic signal: The most notable feature of IL-21 is its powerful synergy with IL-15. Mechanism: IL-21 activates the JAK/STAT pathway (mainly STAT1 and STAT3) through its receptor (IL-21R), while IL-15 activates STAT5. The cooperation of both can more effectively promote proliferation and differentiation of NK cells (Chabab et al., 2020; Waldmann et al., 2020; Wang and Zhao, 2021; Covino et al., 2022). Promotes terminal maturation: IL-21 particularly enhances NK cell expression of CD25 (IL-2Rα), thereby increasing responsiveness to IL-2 and upregulating the expression of cytotoxic molecules (such as perforin and granzyme), making their functionality more mature (Kim et al., 2017).
2. Unique “two-phase” action: promotes expansion vs. limits long-term survival
  Early promotion of expansion: During the early stage of NK cell activation, IL-21 synergizes with IL-15 to effectively promote the proliferation of human NK cells (Rozenberg et al., 2025). Later induction of apoptosis/differentiation: However, prolonged or high concentrations of IL-21 exposure can limit long-term survival and proliferative potential of NK cells by upregulating pro-apoptotic proteins (such as Bim) or inducing terminal differentiation. This is considered a mechanism to prevent excessive immune responses and provide negative feedback regulation. Therefore, the role of IL-21 is to provide “fine-tuned” regulation of NK cell responses: helping to establish a robust and functionally mature NK cell pool in the early phase of the response, and then appropriately “braking” in the later phase to avoid loss of control.
3. Applications in *in vitro* expansion for therapy
  In clinical-grade NK cell *in vitro* expansion culture, the combination of IL-21 + IL-15 has proven to be a very effective regimen, capable of rapidly generating large quantities of highly cytotoxic NK cells. Due to the “restrictive” effect of IL-21, its exposure time and concentration need to be precisely controlled to maximize its proliferative effects while minimizing its pro-apoptotic effects.

Although IL-18 and IL-21 are not “survival-essential” cytokines like IL-15, they play a decisive role in shaping the magnitude, functional quality, and duration of responses of NK cell expansion by providing unique co-stimulatory and regulatory signals. The rational use of the characteristics of IL-18 and IL-21 can better “customize” and optimize the immune response of NK cells in cancer immunotherapy and antiviral immunity.

In addition to their cytotoxic activity, NK cells secrete a variety of cytokines that modulate immune responses. IFN-γ is a key cytokine produced by NK cells, playing a crucial role in antiviral and antitumor immunity. Which also activates macrophages, enhances antigen presentation, and promotes the differentiation of T helper 1 (Th1) cells (Zhang et al., 2019). NK cells also produce other cytokines, such as TNF-α and IL-12, which contribute to inflammation and immune cell recruitment (Mukherjee et al., 2018). The cytokine secretion profile of NK cells can influence the balance between pro-inflammatory and immunosuppressive responses in the tumor microenvironment.

#### 1.2.7 Advantages over T cell responses in early lesion surveillance

NK cells possess unique advantages over T cells in the surveillance and elimination of early viral lesions, particularly in the context of HPV infection (Mandal et al., 2016). Unlike T cells, NK cells can rapidly respond to infected or transformed cells without prior sensitization (Mandal et al., 2016). This allows NK cells to provide immediate protection against early viral lesions before the development of adaptive immune responses (Mandal et al., 2016).

One key advantage of NK cells is their ability to recognize cells that have downregulated MHC-I expression, a common immune evasion strategy employed by HPV-infected cells (Westrich et al., 2019). While T cells require MHC-I to recognize and kill target cells, NK cells can eliminate cells with reduced MHC-I expression through the “missing-self” mechanism (Westrich et al., 2019). This makes NK cells particularly effective at targeting HPV-infected cells that have evaded T cell recognition.

NK cells can infiltrate tissues and recognize cellular stress signals associated with early lesion development (Manjgaladze et al., 2019). They can then eliminate these cells, preventing progression to more advanced disease stages (Manjgaladze et al., 2019). Studies have demonstrated the effectiveness of NK cells in surveilling and eliminating HPV-associated lesions before they progress to cancer (Zhang et al., 2019). For example, a study found that NK cell activity was significantly decreased in the cervix of HPV16-positive patients compared to HPV18-positive patients, suggesting that HPV16 may disable NK cell function in early lesions (Zhang et al., 2019).

### 1.3 Rationale and significance of promoting NK cell activation in HPV-related tumor prevention and treatment

Promoting NK cell activation represents a promising therapeutic strategy for the prevention and treatment of HPV-related tumors (Veluchamy et al., 2017). Enhancing NK cell function can improve antitumor efficacy and prevent tumor development by increasing the elimination of HPV-infected and transformed cells (Veluchamy et al., 2017).

One approach to enhance NK cell activity is through the use of pharmacological agents and innate immune agonists (Cho et al., 2019). Poly-gamma-glutamic acid (γ-PGA) has shown short-term therapeutic effects on cervical intraepithelial neoplasia 1 (CIN 1) and high-risk HPV infection, potentially by modulating immune responses (Cho et al., 2019). Toll-like receptor (TLR) agonists, such as TLR9 agonists, can also stimulate NK cell activity and enhance antitumor immunity (Manjgaladze et al., 2019). The RIG-I agonist M8 triggers cell death and NK cell activation in HPV-associated cancer and potentiates cisplatin cytotoxicity (Girone et al., 2023).

Genetic engineering and cell product optimization offer additional strategies for harnessing NK cells in immunotherapy. Chimeric antigen receptor (CAR)-NK cells can be engineered to express receptors that specifically target tumor-associated antigens, enhancing their specificity and cytotoxicity (Wang et al., 2019; Gong et al., 2021; Rafei et al., 2021). Strategies to enhance NK cell specificity and *in vivo* persistence, such as TGF-β receptor knockout and enhanced CD16 expression, are also being explored (Otegbeye et al., 2018; Wong et al., 2024).

Combination therapies that integrate NK cells with immune checkpoint inhibitors and monoclonal antibodies have shown synergistic effects in preclinical and clinical studies. Cetuximab, an EGFR-targeting monoclonal antibody, can enhance NK cell-mediated ADCC in head and neck squamous cell carcinoma (HNSCC) cell lines (Baysal et al., 2020). Combining NK cell-based therapies with vaccination or conventional treatments may further improve antitumor responses (Amador-Molina et al., 2019).

Targeting NK cells can also address immunoediting and immune evasion seen in HPV-infected tumor environments (William et al., 2021). By restoring MHC-I expression on tumor cells and promoting antigen-specific CD8^+^ T-cell responses, NK cell activation can overcome immune evasion mechanisms and suppress tumor growth (Westrich et al., 2019). The infiltration of CD56^dim^ NK cells is correlated with superior survival in HNSCC, highlighting the importance of NK cells in controlling tumor progression (Mandal et al., 2016).

Collectively, promoting NK cell activation is a rational and significant approach for HPV-related tumor prevention and treatment. Strategies that enhance NK cell function, specificity, and persistence hold great promise for improving outcomes in patients with HPV-associated cancers.

## 2 Biological characteristics of NK cells

### 2.1 NK cell development and differentiation

#### 2.1.1 Origin from bone marrow and maturation pathways

NK cells originate from hematopoietic stem cells (HSCs) in the bone marrow, sharing a common lymphoid progenitor with T and B cells. The development of NK cells is a multistage process, beginning with HSCs differentiating into NK cell precursors. These precursors then mature into immature NK cells before finally becoming fully functional mature NK cells (Luetke-Eversloh et al., 2013).

Key transcription factors play a crucial role in NK cell differentiation. For instance, E4BP4, also known as NFIL3, is essential for the early development of NK cells. EOMES and T-bet are also critical transcription factors that regulate NK cell maturation and function.

Secondary lymphoid tissues, such as lymph nodes and the spleen, serve as important sites for NK cell maturation and education. In these tissues, NK cells acquire their functional competence through interactions with other immune cells and exposure to various cytokines.

#### 2.1.2 Distinction between CD56^+^ and CD56^-^ subsets

NK cells can be broadly divided into two major subsets based on the expression levels of the CD56 marker: CD56^+^ and CD56^-^ (Mandal et al., 2016). These subsets differ significantly in their phenotype, function, and tissue distribution (Mandal et al., 2016).

**Figure 2:**
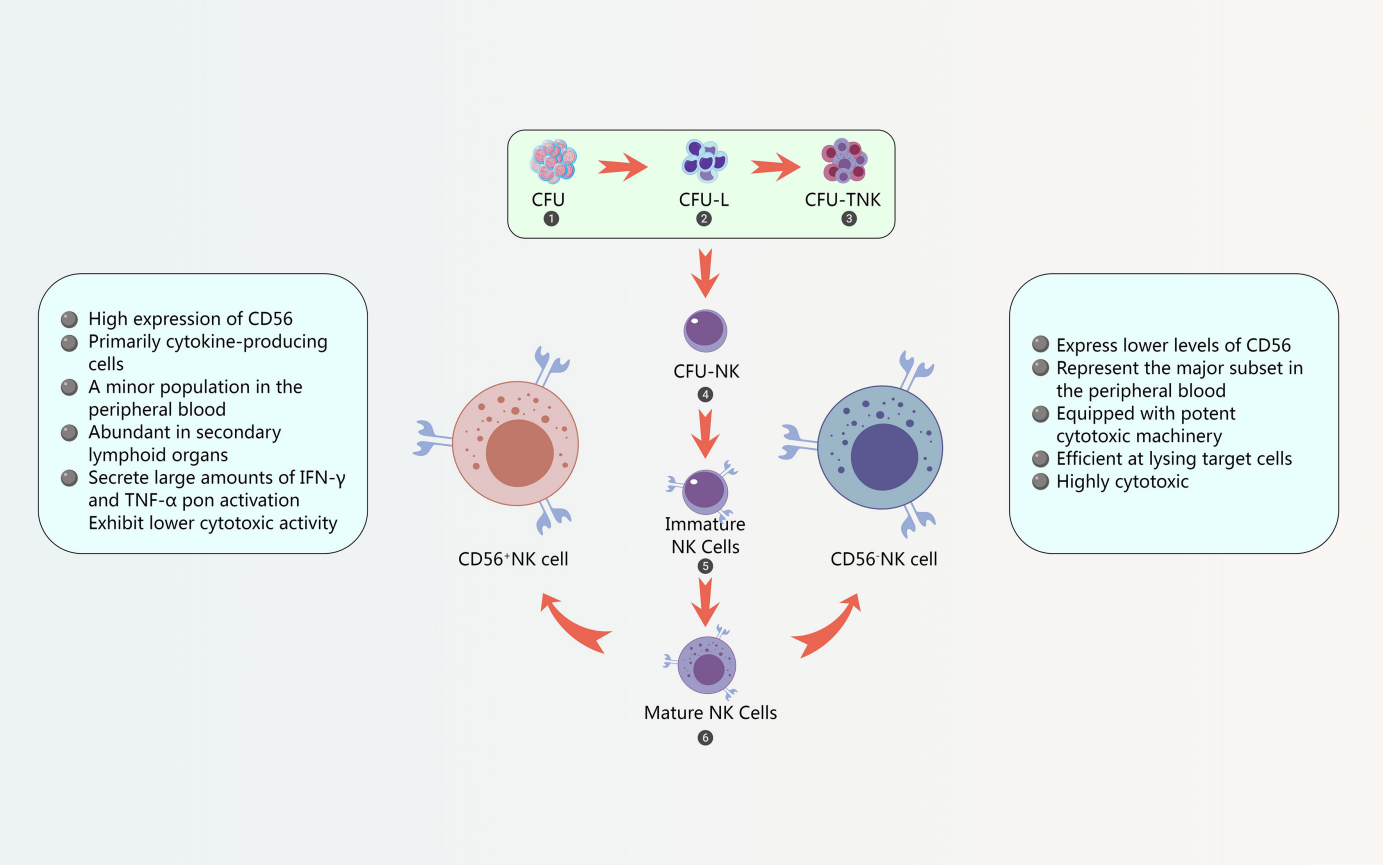
NK cell development and differentiation. The characters of CD56^+^ NK cell and Characters of CD56^-^ NK cells.

CD56^+^ NK cells are characterized by high expression of CD56 and are primarily cytokine-producing cells (Nie et al., 2022). They constitute a minor population in the peripheral blood but are abundant in secondary lymphoid organs (Nie et al., 2022). These cells are known for their ability to secrete large amounts of IFN-γ and TNF-α upon activation, playing a crucial role in immune regulation and antiviral responses. However, CD56^+^ NK cells exhibit lower cytotoxic activity compared to the CD56^-^ subset. Studies have shown that in HPV16 (+) cervical intraepithelial neoplasia (CIN), the number of circulating CD56^+^ NK cells is increased, but their functionality and IFN-γ secretion are reduced (Nie et al., 2022) (**Figure 2**).

CD56^-^ NK cells, on the other hand, express lower levels of CD56 and are highly cytotoxic (Mandal et al., 2016). They represent the major subset in the peripheral blood and are equipped with potent cytotoxic machinery, including perforin and granzymes (Mandal et al., 2016). CD56^-^ NK cells are efficient at lysing target cells, such as virus-infected cells and tumor cells. These cells express high levels of killer cell immunoglobulin-like receptors (KIRs) and CD16 (FcγRIIIa), which mediate ADCC (Mandal et al., 2016). (**Figure 2**).

In HPV-related malignancies, the balance between these subsets can be altered. For instance, a study of HNSCC found that HNSCC had the highest levels of CD56^-^ NK cell infiltration. Understanding the specific roles and alterations of these subsets in the context of HPV-related tumors is essential for developing targeted immunotherapeutic strategies.

### 2.2 Receptor balance and signaling mechanisms

#### 2.2.1 Key activating receptors

**Figure 3:**
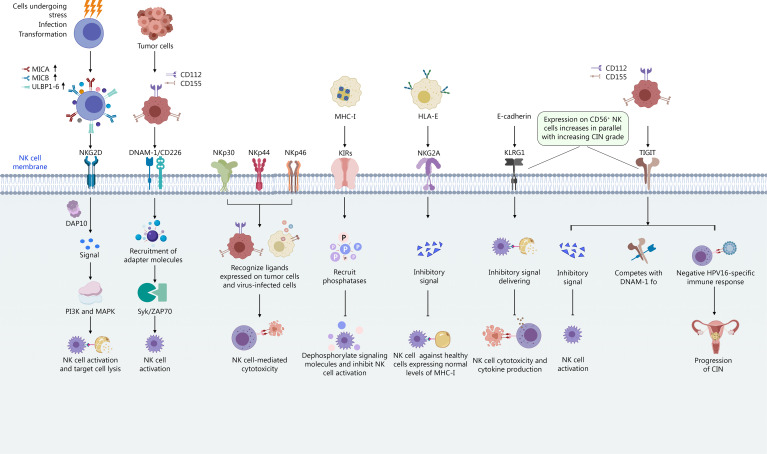
Receptor balance and signaling mechanisms.

NK cell activation is tightly regulated by a balance between activating and inhibitory signals mediated through various cell surface receptors. Activating receptors recognize stress-induced ligands on infected or transformed cells, triggering NK cell effector functions (Espinoza et al., 2016) (**Figure 3**).

NK cells are key components of the innate immune system, renowned for their rapid ability to kill virus-infected cells and tumor cells. Classic NK cell activation markers include: CD69: A very early activation marker expressed within hours after activation. CD25: The alpha chain of the interleukin-2 receptor, indicating the cell’s responsiveness to IL-2, serving as a marker of activation and proliferation. Upregulation of activation receptors: Such as NKG2D, NKp30, NKp44, NKp46, etc. Cytokine production: Such as IFN-γ, TNF-α. Degranulation marker: Membrane expression of CD107a (Shimasaki et al., 2020; Wang et al., 2023).

NKG2D (natural killer group 2, member D) is a key activating receptor expressed on NK cells, as well as some T cell subsets (Espinoza et al., 2016). It recognizes a variety of stress-induced ligands, including MICA, MICB, and ULBP1-6, which are often upregulated on cells undergoing stress, infection, or transformation (Gutiérrez-Hoya et al., 2019). Upon ligand binding, NKG2D signals through the DAP10 adapter protein, activating downstream signaling pathways such as PI3K and MAPK, leading to NK cell activation and target cell lysis (Espinoza et al., 2016). A functional polymorphism in the NKG2D gene, rs1049174, has been shown to modulate NK-cell cytotoxicity and is associated with susceptibility to human papilloma virus-related cancers. Individuals with the low cytotoxicity allele (LNK) exhibit lower NKG2D expression and less efficient NKG2D-mediated functions (Espinoza et al., 2016).

DNAM-1 (DNAX accessory molecule-1), also known as CD226, is another crucial activating receptor on NK cells (Veluchamy et al., 2017). Its ligands include CD112 (nectin-2) and CD155 (PVR, poliovirus receptor), which are adhesion molecules often overexpressed on tumor cells. DNAM-1 signaling involves the recruitment of adapter molecules such as DAP12, leading to the activation of Syk/ZAP70 kinases and subsequent NK cell activation (Veluchamy et al., 2017).

HLA-DR can serve as a late activation marker for NK cells under specific circumstances, but it is neither an early nor the most classic activation marker. Its expression typically indicates that NK cells have undergone intense and sustained activation and have entered a more effector or “memory-like” state. In a resting state, most NK cells do not express or only express low levels of HLA-DR. However, under specific stimuli, NK cells can be induced to express HLA-DR (Erokhina et al., 2018; Kust et al., 2024):

1. Stimulation conditions:
  - Strong cytokine stimulation: Prolonged exposure (usually over 18–24 hours) to high concentrations of IL-2, IL-12, IL-15, and IL-18 (Erokhina et al., 2021).
  - Viral infection: In patients infected with mycobacteria or influenza A, an increased proportion of HLA-DR+ NK cells can be observed (Kust et al., 2021; Harvey et al., 2022).
  - Tumor microenvironment: In the peripheral blood or tumor-infiltrating NK cells of certain cancer patients, there is an increased subpopulation of HLA-DR+ NK cells (Fei et al., 2022).
  - Autoimmune diseases: HLA-DR expression has also been found in NK cells of patients with systemic lupus erythematosus (Cruz-González et al., 2018).
2. Biological significance:
  - Late activation state: HLA-DR expression typically appears later following NK cell activation (24–48 hours), marking the transition from early activation to a sustained, highly activated state. Enhanced effector function: Studies indicate that the HLA-DR+ NK cell subpopulation generally exhibits stronger cytotoxic abilities and the production of IFN-γ (Erokhina et al., 2018; Erokhina et al., 2021).

Associated with “adaptive” or “memory-like” NK cells: Post certain viral infections (especially HCMV), a long-lived subpopulation of NK cells with “memory” properties is induced. These cells often exhibit HLA-DR expression and co-express markers like CD57 and NKG2C, representing a functionally highly specialized NK cell subpopulation (Costa-García et al., 2019; Kobyzeva et al., 2020).

Unknown function of reverse signaling: Although NK cells express HLA class II molecules, they typically do not express classic co-stimulatory molecules. The function of these HLA-DR molecules on NK cells is not fully understood, with hypotheses suggesting they may receive “reverse signals” through interaction with CD4+ T cells, thereby regulating NK cell function or survival, though further research is needed to confirm this. In flow cytometry analysis, HLA-DR can serve as a useful marker to identify and sort NK cell subpopulations that have undergone strong, sustained activation. In complex samples (such as peripheral blood mononuclear cells), HLA-DR is often used to differentiate NK cells from monocytes/dendritic cells. A common gating strategy is: CD3- CD19- CD14- CD56+ HLA-DR- to define “classical” resting NK cells. Therefore, when HLA-DR expression is observed on CD56+ cells, it indicates an activated subpopulation.

Other activating receptors include the natural cytotoxicity receptors (NCRs): NKp30, NKp44, and NKp46. These receptors recognize ligands expressed on tumor cells and virus-infected cells, contributing to NK cell-mediated cytotoxicity (Gutiérrez-Hoya et al., 2019).

#### 2.2.2 Inhibitory receptors

Inhibitory receptors play a critical role in preventing autoimmunity and maintaining NK cell tolerance. These receptors recognize MHC-I molecules on healthy cells, delivering inhibitory signals that counteract activating signals and prevent NK cell activation (**Figure 3**).

Killer cell immunoglobulin-like receptors (KIRs) are a diverse family of inhibitory receptors that recognize specific HLA-A, -B, and -C alleles (Luetke-Eversloh et al., 2013). KIRs are highly polymorphic, and their expression varies among individuals, contributing to the heterogeneity of NK cell responses. Upon binding to MHC-I, KIRs recruit phosphatases such as SHP-1, which dephosphorylate signaling molecules and inhibit NK cell activation (Pende et al., 2019; Sivori et al., 2019; Wu et al., 2021).

NKG2A is another important inhibitory receptor that forms a heterodimer with CD94 and recognizes HLA-E. HLA-E presents peptides derived from the signal sequences of other MHC-I molecules, serving as a sensor of MHC-I expression. Engagement of NKG2A by HLA-E delivers an inhibitory signal, preventing NK cell activation against healthy cells expressing normal levels of MHC-I (Costa-García et al., 2019; Kobyzeva et al., 2020).

TIGIT (T cell immunoreceptor with Ig and ITIM domains) is an inhibitory receptor expressed on NK cells and T cells. Its ligands include CD155 (PVR) and CD112 (Nectin-2), which are also ligands for the activating receptor DNAM-1 (Nie et al., 2022). TIGIT competes with DNAM-1 for ligand binding, delivering an inhibitory signal that suppresses NK cell activation. Upregulation of TIGIT and its ligands may negatively regulate cervical CD56^bright^ NK-mediated immunity to HPV16 and contribute to the progression of CIN (Nie et al., 2022).

KLRG1 (killer cell lectin-like receptor G1) is another inhibitory receptor expressed on NK cells and T cells, often associated with cellular senescence and exhaustion (Zhang et al., 2024). Its ligand is E-cadherin, an adhesion molecule expressed on epithelial cells. KLRG1 engagement delivers an inhibitory signal, suppressing NK cell cytotoxicity and cytokine production. Similar to TIGIT, KLRG1 expression on CD56^bright^ NK cells increases in parallel with increasing CIN grade, suggesting a role in immune evasion.

HPV may exploit these inhibitory pathways to evade NK cell surveillance. For example, HPV-infected cells may downregulate MHC-I expression to avoid T cell recognition but upregulate TIGIT and KLRG1 ligands to inhibit NK cell activation (Nie et al., 2022; Nie et al., 2022; Zhang et al., 2024).

## 3 Interaction between HPV and NK cells

### 3.1 Impact of HPV infection on NK cell numbers and functionality

#### 3.1.1 Reduction in local NK cell infiltration and activity in HPV-infected lesions

**Figure 4:**
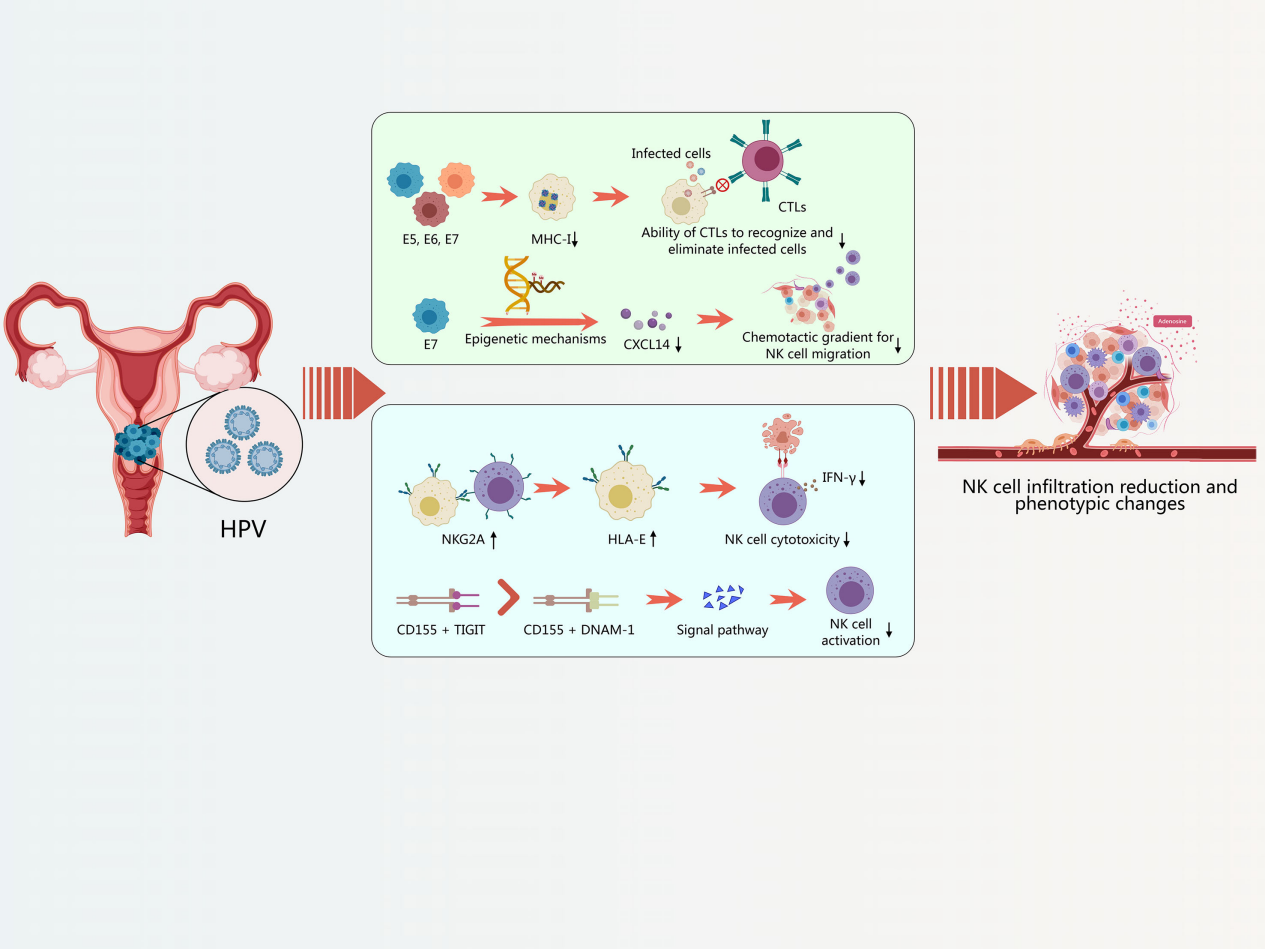
Interaction between HPV and NK cells. Viral oncoproteins downregulate the expression of MHC-I; Upregulation of inhibitory molecules on NK cells.

HPV infection, a primary cause of cervical cancer and a growing contributor to head and neck cancers, significantly impacts the tumor microenvironment, particularly affecting the infiltration and activity of NK cells (Westrich et al., 2019; Soleymaninejadian et al., 2022). Clinical and experimental evidence consistently demonstrates a reduction in NK cell infiltration within HPV-associated lesions, such as cervical intraepithelial neoplasia (CIN) and HNSCC (Westrich et al., 2019; Zhang et al., 2019) (**Figure 4**).

Studies have shown that in HPV-infected cervical tissues, there is a notable decrease in the number of NK cells present compared to healthy tissues or tissues infected with other HPV types (Zhang et al., 2019). This reduction in NK cell infiltration is crucial because NK cells are vital for early detection and elimination of virally infected and transformed cells. The decreased presence of NK cells impairs the local immune surveillance, facilitating HPV persistence and disease progression.

Several mechanisms may account for the diminished NK cell infiltration in HPV-infected lesions. One prominent factor is the impaired chemokine signaling, particularly the downregulation of CXCL14, a chemokine known for its role in recruiting NK cells and T cells to the tumor microenvironment (Cicchini et al., 2016; Westrich et al., 2019; Westrich et al., 2020). CXCL14 expression is often suppressed by the HPV oncoprotein E7, leading to reduced recruitment of NK cells to the infected site. Without sufficient CXCL14, the chemotactic gradient necessary for NK cell migration is weakened, resulting in fewer NK cells reaching the lesion (Cicchini et al., 2016; Westrich et al., 2019; Westrich et al., 2020).

Altered expression of adhesion molecules also contributes to the reduced NK cell infiltration. Adhesion molecules are crucial for NK cells to adhere to and migrate through the endothelium into the infected tissue. HPV infection can disrupt the normal expression patterns of these molecules, hindering the ability of NK cells to effectively infiltrate the lesion (Gutiérrez-Hoya and Soto-Cruz, 2021; O'Hara et al., 2024).

Comparative studies between HPV-positive and HPV-negative lesions further highlight the differences in NK cell activity (Evans et al., 2022). HPV-positive lesions often exhibit lower NK cell cytotoxicity and IFN-γ production compared to HPV-negative lesions (Zhang et al., 2019). This suggests that even when NK cells are present in HPV-infected tissues, their ability to eliminate infected cells is compromised, contributing to the immune evasion strategies employed by HPV (**Figure 4**).

#### 3.1.2 Evidence of NK cell exhaustion and altered receptor expression

Beyond the reduction in NK cell numbers, HPV infection induces phenotypic changes in NK cells, indicative of exhaustion and altered receptor expression (Soleymaninejadian et al., 2022). NK cell exhaustion is characterized by the upregulation of inhibitory receptors and downregulation of activating receptors, leading to impaired functionality (Mandal et al., 2016).

Exhaustion markers, such as PD-1 (programmed cell death protein 1) and TIM-3 (T-cell immunoglobulin and mucin-domain containing-3), are often found to be upregulated on NK cells in HPV-infected tissues (Mandal et al., 2016; Hsu et al., 2018; Pesce et al., 2019; Wang et al., 2025). The expression of these markers indicates that NK cells have been chronically stimulated and are functionally impaired. PD-1, for example, binds to its ligand PD-L1, which is often overexpressed in tumors, delivering an inhibitory signal that suppresses NK cell activity.

Furthermore, HPV infection leads to shifts in the expression of activating and inhibitory receptors on NK cells. Activating receptors like NKG2D and DNAM-1 are crucial for recognizing and eliminating infected or transformed cells (Mariuzza et al., 2025). However, in HPV-infected environments, the expression of NKG2D ligands, such as MICA/B, can be downregulated by HPV oncoproteins, reducing NK cell activation (Koneva et al., 2018; Gutiérrez-Hoya et al., 2019). Similarly, DNAM-1 ligands may also be reduced, further impairing NK cell-mediated cytotoxicity.

Conversely, the expression of inhibitory receptors, including KIRs, NKG2A, TIGIT, and KLRG1, is often increased on NK cells in HPV-infected tissues (Mandal et al., 2016). These inhibitory receptors bind to ligands on target cells, delivering inhibitory signals that dampen NK cell activity. For instance, NKG2A binds to HLA-E, which can be upregulated in HPV-infected cells, leading to NK cell inhibition (Gutiérrez-Hoya et al., 2019). TIGIT, another inhibitory receptor, interacts with CD155, which is also frequently overexpressed in tumors, suppressing NK cell function (Mandal et al., 2016; Chauvin et al., 2020; Harjunpää and Guillerey, 2020; Mu and Guan, 2024; Sun et al., 2025). KLRG1, associated with replicative senescence, is upregulated in exhausted NK cells, further reducing their cytotoxic potential (Zhang et al., 2019).

*In vitro* and *in vivo* studies have consistently demonstrated the functional impairment of NK cells in the context of HPV infection. These studies show that NK cells from HPV-infected tissues exhibit reduced cytotoxicity and IFN-γ production upon stimulation (Zhang et al., 2019). The decreased IFN-γ production is particularly significant, as IFN-γ is a key cytokine involved in activating other immune cells and promoting anti-tumor responses. The cumulative effect of these phenotypic and functional changes leads to a weakened NK cell response, facilitating HPV persistence and the development of HPV-associated cancers.

### 3.2 HPV-mediated immune evasion mechanisms

#### 3.2.1 Downregulation of MHC-I and chemokine signaling

To establish persistent infections and promote tumorigenesis, HPV employs several strategies to evade immune surveillance, particularly targeting NK cell recognition and recruitment (Soleymaninejadian et al., 2022). Two critical mechanisms involve the downregulation of MHC-I molecules and the suppression of chemokine production, such as CXCL14 (Cicchini et al., 2016; Westrich et al., 2019; Westrich et al., 2020).

MHC-I molecules play a central role in presenting intracellular antigens to CD8^+^ T cells, initiating an adaptive immune response. However, downregulation of MHC-I is a common immune evasion strategy used by viruses and tumors to avoid recognition by CTLs. This downregulation also affects NK cell recognition through the “missing-self hypothesis,” which posits that NK cells are activated when they encounter cells with reduced MHC-I expression (Gutiérrez-Hoya et al., 2019). While NK cells are generally activated by the absence of MHC-I, HPV-infected cells can exploit this mechanism to their advantage.

HPV-infected cells can downregulate MHC-I expression through various mechanisms, including the action of viral oncoproteins like E5, E6, and E7 (Soleymaninejadian et al., 2022). These oncoproteins interfere with the expression and cell surface presentation of MHC-I molecules, reducing the ability of CTLs to recognize and eliminate infected cells. Paradoxically, while the missing-self hypothesis suggests that decreased MHC-I should activate NK cells, HPV-infected cells often upregulate inhibitory ligands that counteract this activation, maintaining NK cell quiescence (Gutiérrez-Hoya et al., 2019).

In addition to MHC-I downregulation, HPV suppresses the production of chemokines, such as CXCL14, further impairing NK cell recruitment. CXCL14 is a homeostatic chemokine that plays a crucial role in attracting NK cells and T cells to the tumor microenvironment (Cicchini et al., 2016; Westrich et al., 2019). HPV oncoproteins, particularly E7, downregulate CXCL14 expression through epigenetic mechanisms, such as DNA hypermethylation of the CXCL14 promoter (Cicchini et al., 2016). This epigenetic silencing reduces the production and secretion of CXCL14, weakening the chemotactic gradient needed for NK cell migration to the infected site.

Studies have demonstrated that restoring CXCL14 expression in HPV-positive cancer cells can reverse immune suppression and promote tumor clearance (Cicchini et al., 2016; Westrich et al., 2019). *In vivo* experiments using mouse models have shown that CXCL14 re-expression significantly increases NK cell and T cell infiltration into tumors, leading to enhanced anti-tumor immunity and improved survival (Cicchini et al., 2016; Westrich et al., 2019). These findings underscore the importance of CXCL14 in mediating NK cell recruitment and highlight the impact of HPV-mediated chemokine suppression on immune evasion.

Modulation of chemokine pathways represents a potential therapeutic strategy to enhance NK cell function in HPV-associated cancers. By restoring the production or activity of chemokines like CXCL14, it may be possible to improve NK cell infiltration and anti-tumor responses, ultimately overcoming HPV-mediated immune evasion.

#### 3.2.2 Upregulation of inhibitory molecules on NK cells

HPV-infected cells and the surrounding tumor microenvironment not only downregulate activating signals but also actively upregulate inhibitory ligands that engage NK cell inhibitory receptors, further suppressing NK cell activity (Soleymaninejadian et al., 2022). Key inhibitory molecules involved in this process include HLA-E, which binds to NKG2A, and CD155, which interacts with TIGIT (Mandal et al., 2016; Gutiérrez-Hoya et al., 2019; Chauvin et al., 2020; Harjunpää and Guillerey, 2020; Mu and Guan, 2024; Sun et al., 2025).

HLA-E is a non-classical MHC-I molecule that presents peptides derived from the signal sequence of other MHC-I molecules. In HPV-infected cells, HLA-E expression is often upregulated, providing a ligand for the inhibitory receptor NKG2A on NK cells. The interaction between HLA-E and NKG2A delivers a potent inhibitory signal, dampening NK cell cytotoxicity and IFN-γ production (Cicchini et al., 2017; Gutiérrez-Hoya et al., 2019; Franciosi et al., 2020; Carrette and Vivier, 2023). This mechanism is particularly effective because it exploits the missing-self response; even when MHC-I is downregulated, the upregulation of HLA-E can still inhibit NK cell activation.

CD155, also known as PVR (poliovirus receptor), is another inhibitory ligand that is frequently overexpressed in HPV-infected cells and tumor cells (Mandal et al., 2016). CD155 binds to several receptors on NK cells, including the activating receptor DNAM-1 and the inhibitory receptor TIGIT. While DNAM-1 promotes NK cell activation, the upregulation of CD155 in the tumor microenvironment preferentially engages TIGIT, delivering an inhibitory signal that suppresses NK cell activity (Mandal et al., 2016).

TIGIT is an immune checkpoint receptor expressed on NK cells and T cells. It competes with the activating receptor CD226 (DNAM-1) for binding to CD155 (Wang et al., 2019; Long et al., 2024). When TIGIT binds to CD155, it inhibits NK cell function by suppressing cytotoxicity and cytokine production. The balance between TIGIT and DNAM-1 signaling is crucial for regulating NK cell responses, and HPV-infected cells can manipulate this balance to their advantage by upregulating CD155 and promoting TIGIT engagement (Chauvin et al., 2020; Harjunpää and Guillerey, 2020; Nie et al., 2022; Mu and Guan, 2024; Sun et al., 2025).

KLRG1 is another inhibitory receptor upregulated on NK cells in the context of chronic viral infections and cancer (Mandal et al., 2016). KLRG1 is a marker of terminal differentiation and replicative senescence in NK cells, indicating that these cells have undergone repeated stimulation and are functionally exhausted. The upregulation of KLRG1 is associated with reduced proliferative capacity and decreased cytotoxicity, further impairing the ability of NK cells to control HPV infection (Nie et al., 2022; Zhang et al., 2024).

Therapeutic strategies targeting these inhibitory pathways are being explored to enhance NK cell activity in HPV-associated cancers. Checkpoint blockade, using antibodies to block the interaction between inhibitory receptors and their ligands, can restore NK cell function and promote anti-tumor responses (Mandal et al., 2016). For example, anti-TIGIT antibodies can block the interaction between TIGIT and CD155, freeing DNAM-1 to engage CD155 and activate NK cells. Similarly, blocking the interaction between NKG2A and HLA-E can enhance NK cell cytotoxicity against HPV-infected cells.

### 3.3 Molecular modulation by HPV oncoproteins

#### 3.3.1 Direct and indirect effects of E6/E7 on NK cell receptor-ligand interactions

HPV oncoproteins, primarily E6 and E7, play a central role in modulating the tumor microenvironment and suppressing NK cell function through both direct and indirect mechanisms (Cicchini et al., 2016; Soleymaninejadian et al., 2022). These oncoproteins interfere with NK cell receptor-ligand interactions, alter cytokine production, and promote immune evasion, ultimately contributing to HPV persistence and cancer progression.

One direct effect of E6 and E7 is the alteration of NK cell receptor ligands on the surface of HPV-infected cells. For example, E6 can induce the proteasomal degradation of MICA and MICB, ligands for the activating receptor NKG2D (Gutiérrez-Hoya et al., 2019). By reducing the expression of these ligands, E6 impairs the ability of NK cells to recognize and eliminate HPV-infected cells. This downregulation of NKG2D ligands is a critical mechanism by which HPV evades NK cell-mediated immune surveillance.

In addition to directly affecting receptor ligands, E6 and E7 also have indirect effects on NK cell function through the modulation of cytokine production. HPV infection can induce the production of immunosuppressive cytokines, such as TGF-β and IL-10, which suppress NK cell activity (Mukherjee et al., 2018; Wang et al., 2022). TGF-β, for instance, inhibits NK cell cytotoxicity and IFN-γ production, while IL-10 suppresses the expression of activating receptors and promotes NK cell exhaustion.

E6 and E7 can also indirectly affect NK cell function by altering the expression of MHC-I molecules and chemokines. HPV-mediated downregulation of MHC-I reduces NK cell activation through the missing-self hypothesis, while suppression of chemokines like CXCL14 impairs NK cell recruitment to the tumor microenvironment (Cicchini et al., 2016; Westrich et al., 2019). These indirect effects further contribute to the overall suppression of NK cell responses in HPV-infected tissues.

#### 3.3.2 Consequences for tumor microenvironment remodeling and malignant transformation

HPV-mediated NK cell dysfunction has significant consequences for tumor microenvironment remodeling and malignant transformation (Soleymaninejadian et al., 2022). By suppressing NK cell activity, HPV creates an immunosuppressive environment that favors tumor progression and immune evasion. This allows HPV-infected cells to proliferate unchecked and evade immune destruction, increasing the risk of malignant transformation.

The persistent suppression of NK cells in the tumor microenvironment also contributes to the recruitment and activation of other immunosuppressive cells, such as Tregs and MDSCs (Hamilton and Plangger, 2021; Fantini et al., 2023). Tregs suppress the activity of effector T cells and NK cells, while MDSCs inhibit T cell proliferation and promote immune tolerance. The combined effect of these immunosuppressive cells further weakens the anti-tumor immune response, facilitating tumor growth and metastasis.

In persistent HPV infections, the chronic suppression of NK cell function is linked to an increased risk of malignant transformation. Studies have shown that individuals with impaired NK cell activity are more likely to develop HPV-associated cancers, such as cervical cancer and head and neck cancer (Zhang et al., 2019). This highlights the importance of NK cells in controlling HPV infection and preventing the development of cancer.

**Figure 5:**
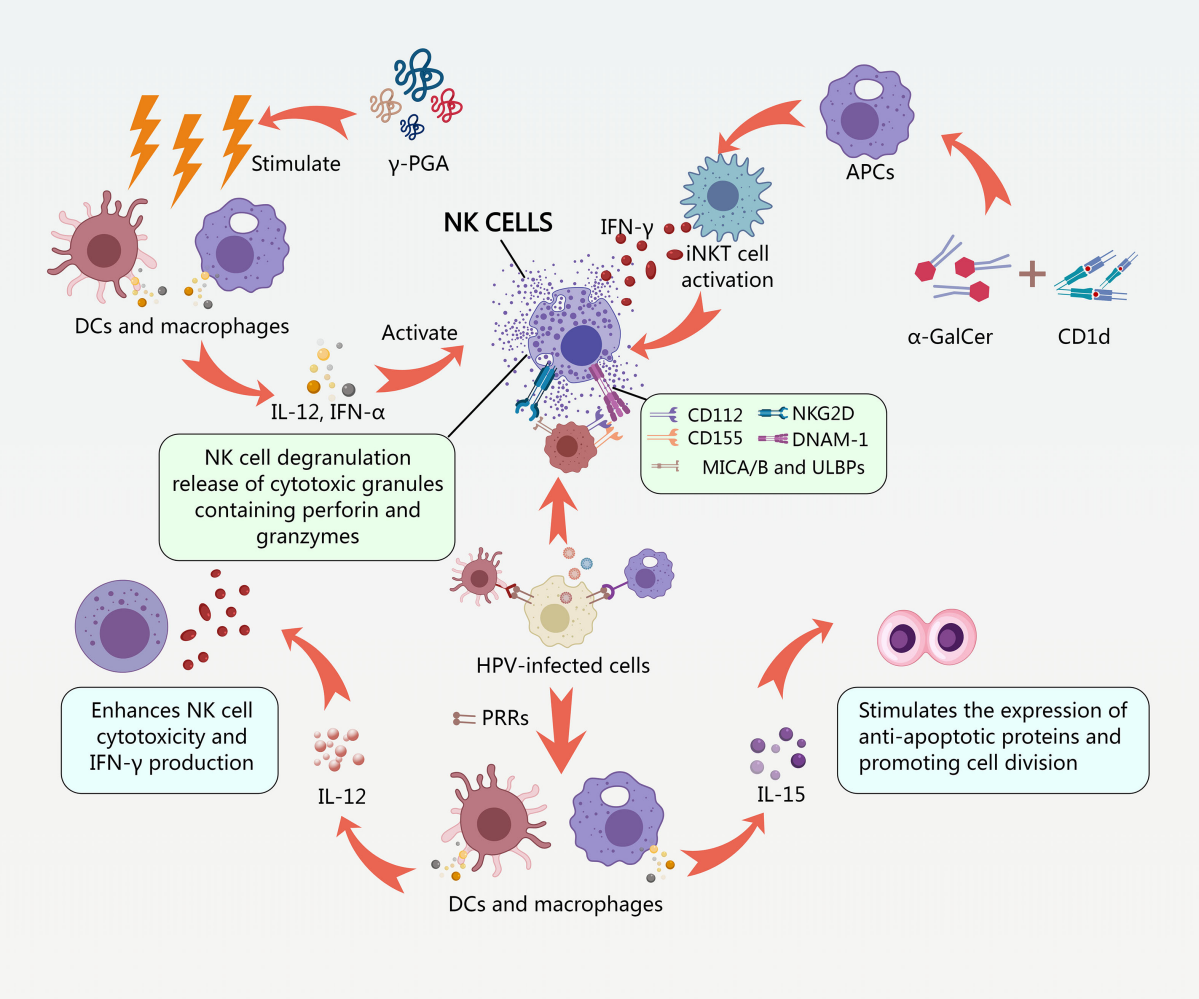
NK cell activation mechanisms and intervention strategies.

Therapeutic interventions aimed at reversing HPV-mediated NK cell dysfunction represent a promising strategy for preventing and treating HPV-associated cancers. Cytokine therapy, using IL-12 or IL-15, can enhance NK cell activity and promote anti-tumor responses (Mukherjee et al., 2018). Adoptive NK cell transfer, in which NK cells are expanded *ex vivo* and then infused back into the patient, can also restore NK cell function and promote tumor regression (Mariuzza et al., 2025). These interventions have shown promise in preclinical and clinical studies, suggesting that they may be effective in reversing the immunosuppressive effects of HPV and preventing malignant transformation (**Figure 5**).

## 4 NK cell activation mechanisms and intervention strategies

### 4.1 Endogenous activation pathways

#### 4.1.1 Role of NK cell receptor-ligand interactions

NK cell activation is tightly regulated by a balance between activating and inhibitory signals transduced through various cell surface receptors (Zhang et al., 2019). Activating receptors such as NKG2D and DNAM-1 play a crucial role in recognizing stress-induced ligands on HPV-infected or transformed cells (Zhang et al., 2019). NKG2D, for instance, recognizes a range of ligands including MICA, MICB, and ULBPs, which are often upregulated on cells experiencing cellular stress, DNA damage, or viral infection (Gutiérrez-Hoya et al., 2019). DNAM-1 interacts with ligands such as PVR (CD155) and Nectin-2 (CD112), which are also frequently overexpressed on tumor cells (Zhang et al., 2019) (**Figure 5**).

HPV oncoproteins, particularly E6 and E7, can modulate the expression of these ligands, thus affecting NK cell detection (Cicchini et al., 2016). For example, HPV E7 has been shown to downregulate MHC-I expression, which can indirectly affect the presentation of NKG2D ligands (Westrich et al., 2019). Additionally, E6 and E7 can directly interfere with the signaling pathways that regulate the expression of MICA/B and ULBPs, leading to immune evasion (Cicchini et al., 2016).

Studies have demonstrated altered NKG2D ligand expression in HPV-associated lesions. Cervical cancer cells, for example, often express MICA/B, facilitating NK cell recognition and potential lysis (Gutiérrez-Hoya et al., 2019). However, this expression can be variable, and in some cases, HPV-infected cells downregulate these ligands to evade immune surveillance (Cicchini et al., 2016). CXCL14, a chemokine downregulated by HPV oncoprotein E7, can restore MHC-I expression on HPV-positive head and neck cancer cells, promoting antigen-specific CD8^+^ T-cell responses (Westrich et al., 2019). This restoration of MHC-I expression is crucial for effective antitumor immunity (Westrich et al., 2019).

The functional consequences of receptor-ligand interactions on NK cell cytotoxicity and cytokine production are significant. Engagement of activating receptors like NKG2D and DNAM-1 triggers NK cell degranulation, leading to the release of cytotoxic granules containing perforin and granzymes, which induce target cell apoptosis (Soleymaninejadian et al., 2022). These interactions also stimulate the production of IFN-γ, a crucial cytokine that enhances the antitumor response by activating other immune cells and promoting tumor cell apoptosis (Mukherjee et al., 2018).

#### 4.1.2 Influence of pro-inflammatory cytokines

Pro-inflammatory cytokines such as IL-15 and IL-12 are vital in enhancing NK cell proliferation, survival, and effector functions (Mukherjee et al., 2018). These cytokines promote the activation of signaling pathways within NK cells, leading to increased cytotoxicity and cytokine production (Mukherjee et al., 2018). IL-15 is essential for NK cell survival and homeostasis, stimulating the expression of anti-apoptotic proteins and promoting cell division (Mukherjee et al., 2018). IL-12 enhances NK cell cytotoxicity and IFN-γ production, playing a critical role in bridging innate and adaptive immunity (Mukherjee et al., 2018).

Dendritic cells (DCs) and macrophages are key players in secreting these cytokines in response to HPV infection (Xu et al., 2020; Boia et al., 2023). Upon encountering HPV-infected cells, DCs and macrophages are activated through pattern recognition receptors (PRRs) such as Toll-like receptors (TLRs), leading to the production of IL-12 and IL-15 (Hopcraft and Damania, 2017; Manjgaladze et al., 2019). These cytokines then activate NK cells, initiating an early immune response against the viral infection (**Figure 5**).

Dysregulation of cytokine signaling in HPV-related tumors can significantly impact NK cell activity. The TME often contains immunosuppressive factors that inhibit the production or activity of IL-12 and IL-15, leading to impaired NK cell function (Luetke-Eversloh et al., 2013). For instance, certain tumor-derived factors can promote the differentiation of Tregs and MDSCs, which suppress NK cell activity through the production of immunosuppressive cytokines such as IL-10 and TGF-β (Luetke-Eversloh et al., 2013) (**Figure 5**).

Preclinical and clinical studies have demonstrated cytokine-based NK cell activation strategies. IL-15 super-agonists, for example, have shown promise in enhancing NK cell activity and improving antitumor responses in various cancers, including HPV-related malignancies (Mukherjee et al., 2018). Clinical trials evaluating the efficacy of IL-12 and IL-15 in combination with other immunotherapeutic agents are ongoing, with the goal of enhancing NK cell-mediated tumor control (Mukherjee et al., 2018).

### 4.2 Exogenous modulators of NK cell activity

#### 4.2.1 Pharmacological agents

Pharmacological agents can stimulate NK cells through direct or indirect mechanisms, enhancing their ability to target and eliminate HPV-infected cells. Poly-γ-glutamic acid (γ-PGA) and α-galactosylceramide (α-GalCer) are two examples of such agents (Amador-Molina et al., 2019; Cho et al., 2019).

γ-PGA is a natural polymer that has shown promise in enhancing immune responses. While γ-PGA itself does not directly activate NK cells, it can stimulate DCs and macrophages, leading to the production of cytokines such as IL-12 and IFN-α, which in turn activate NK cells (Cho et al., 2019) (**Figure 5**). In a randomized, double-blind, phase II trial, oral administration of γ-PGA to women with CIN 1 resulted in a significantly higher histologic regression rate compared to the control group. The γ-PGA group also showed a significant increase in HPV clearance (Cho et al., 2019; Kim et al., 2019).

α-GalCer is a glycolipid that stimulates NK cells via the activation of invariant natural killer T (iNKT) cells. α-GalCer binds to the CD1d molecule on antigen-presenting cells (APCs), leading to the activation of iNKT cells. Activated iNKT cells then release large amounts of cytokines, including IFN-γ, which activates NK cells (Amador-Molina et al., 2019). Vaccination with HPV-18 E1 protein plus α-GalCer in a mouse model induced CD8^+^ cytotoxic responses and impaired the growth of E1-expressing tumors, demonstrating the potential of α-GalCer as an adjuvant for HPV vaccines (Amador-Molina et al., 2019; Amador-Molina et al., 2019) (**Figure 5**).

These agents have the potential to overcome HPV-mediated immune suppression by enhancing NK cell activity and promoting antitumor immunity. Preclinical and clinical trial data support their use in HPV-related malignancies. For instance, γ-PGA has shown therapeutic effects on CIN 1 and high-risk HPV infection, making it a promising non-invasive oral medication for women with these conditions (Cho et al., 2019).

#### 4.2.2 Innate immune agonists (STING, RIG-I Agonists)

Innate immune agonists, such as STING (Stimulator of Interferon Genes) and RIG-I (Retinoic acid-inducible gene I) agonists, are potent modulators of NK cell activity. These agonists activate NK cells by inducing type I interferon responses and other pro-inflammatory cytokines (Girone et al., 2023; Mandracci et al., 2025).

STING agonists activate the STING pathway, leading to the production of type I interferons (IFN-α/β) and other cytokines that stimulate NK cell activation, proliferation, and cytotoxicity (Girone et al., 2023). However, HPV-associated malignancies often exhibit a severely impaired cGAS-STING axis, limiting the effectiveness of STING agonists in these tumors (Girone et al., 2023).

RIG-I agonists, on the other hand, activate NK cells by binding to RIG-I, a cytoplasmic RNA helicase that recognizes viral RNA. Activation of RIG-I leads to the production of type I interferons and other pro-inflammatory cytokines, which enhance NK cell-mediated clearance of HPV-infected cells (Daßler-Plenker et al., 2019; Girone et al., 2023). The RIG-I agonist M8 has been shown to trigger cell death and NK cell activation in HPV-associated cancer cells and potentiate cisplatin cytotoxicity. M8 transfection of cervical carcinoma-derived cell lines (CaSki and HeLa) triggers intrinsic apoptotic cell death and enhances cisplatin-mediated cell killing in a RIG-I dependent manner (Girone et al., 2023). In a syngeneic mouse model of HPV16-driven cancer, M8 treatment reduced tumor growth and increased the number of activated NK cells in the tumor microenvironment (Girone et al., 2023).

Ongoing clinical trials are evaluating STING and RIG-I agonists in combination with NK cell therapies. Given the highly conserved molecular mechanisms of carcinogenesis and genomic features of HPV-driven cancers, targeting RIG-I may represent an effective immunotherapeutic strategy in this setting, favoring the development of de-escalating strategies (Girone et al., 2023).

### 4.3 Genetic engineering and cell product optimization

#### 4.3.1 Development of CAR-NK cells and iPSC-derived “off-the-shelf” NK products

**Figure 6:**
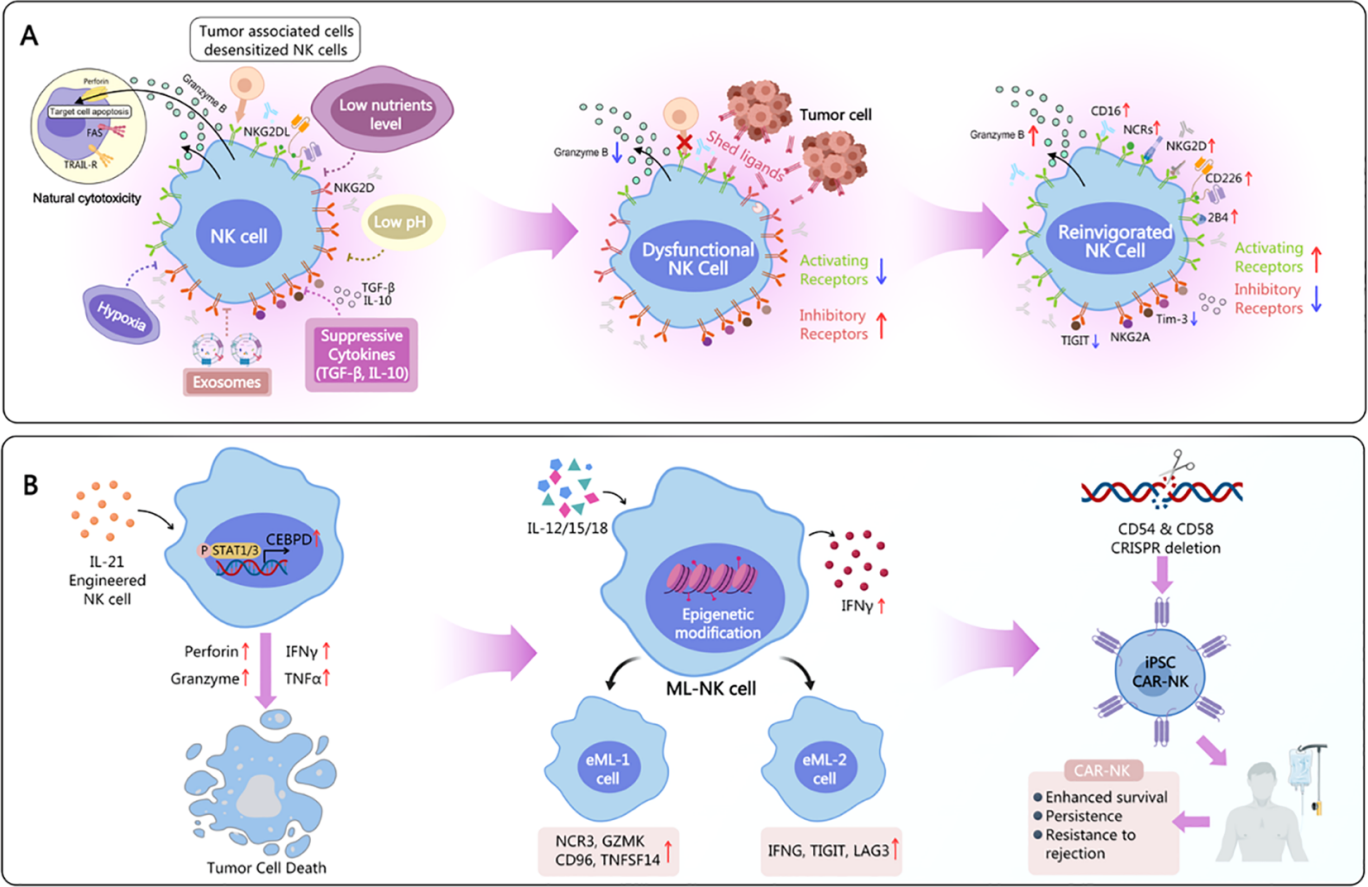
Regulatory targets for NK cells in cancer therapy and disability, as well as engineering modifications of NK cells. **(A)** Mechanisms by which tumor-associated cells affect the anticancer abilities of NK cells, leading to disability and potential regulatory patterns induced by re-therapy. **(B)** Engineering modifications of NK cells, such as IL-21 modification, ML-NK cell preparation, and iPSC-CAR-NK modification to enhance the anticancer activity of NK cells.

Genetic engineering has opened new avenues for enhancing NK cell efficacy against HPV-related tumors. CAR-NK cells and iPSC-derived NK cells represent promising strategies in this regard (Li et al., 2018; Xie et al., 2020; Goldenson et al., 2022; Zhang et al., 2022; Lin et al., 2023) (**Table 2**, **Figure 6**).

**Table 2:** Modification culture and characteristics of NK cells.

Characteristics	Primary NK cells (Zhu and Kaufman, 2019)	CAR-NK (Xie et al., 2020; Peng et al., 2024)	iPSC-NK (Li et al., 2018)	CAR-iPSC-NK (Lin et al., 2023)
Source	Donor Peripheral Blood/Cord Blood	Donor Peripheral Blood/Cord Blood/NK92 Cell Line	iPSC Cell Bank	Genetically Engineered iPSC Cell Bank
Scalability	Limited	Moderate	Unlimited	Unlimited
Uniformity	Poor (donor-dependent)	Moderate	High	High
Engineering Difficulty	Difficult (difficult to transfect primary cells)	Difficult	Easy (manipulation at stem cell stage)	Easy (manipulation at stem cell stage)
Core Advantages	High safety, multiple killing	Strong, targeting, high safety	Scalable, uniform quality	Targeting + scalability + uniform quality + enhanced function
Main Challenges	Quantity, heterogeneity, persistence	Manufacturing process, persistence	Optimization of differentiation protocols, tumorigenicity risk	Long-term safety validation, regulatory approval

CAR-NK cells are engineered to express a chimeric antigen receptor (CAR) that targets specific antigens on tumor cells. The CAR typically consists of an extracellular antigen-binding domain (e.g., a single-chain variable fragment, scFv), a transmembrane domain, and intracellular signaling domains that activate NK cells upon antigen recognition (Gong et al., 2021). CAR-NK cells can be designed to target HPV oncoproteins (e.g., E6/E7) or tumor-associated antigens expressed on HPV-infected cells (Poorebrahim et al., 2023) (**Figure 6B**).

Induced pluripotent stem cells (iPSC)-derived NK cells offer several advantages over primary NK cells, including scalability and reduced alloreactivity. iPSCs can be generated from various cell types and differentiated into NK cells *in vitro*. iPSC-derived NK cells can be produced in large quantities, providing an “off-the-shelf” product for adoptive cell therapy. Moreover, iPSC-derived NK cells can be genetically modified to enhance their antitumor activity (Li et al., 2018; Cichocki et al., 2020; Woan et al., 2021; Goldenson et al., 2022; Maddineni et al., 2022) (**Figure 6B**).

Preclinical studies have demonstrated the efficacy of CAR-NK cells and iPSC-derived NK cells against HPV-related tumors. CAR-NK cells targeting HPV E7, for instance, have shown potent cytotoxicity against HPV-positive cervical cancer cells *in vitro* and *in vivo* (Kutle et al., 2024; Polten et al., 2025). iPSC-derived NK cells have also exhibited promising antitumor activity in preclinical models of HPV-related malignancies (Wei et al., 2025). However, clinical translation faces challenges, including optimizing CAR design, enhancing NK cell persistence *in vivo*, and addressing potential off-target effects.

A detailed introduction to the relevant CAR-NK cell therapy and iPSC-induced NK cell technology in clinical trials. Both are the most advanced and promising directions in today’s field of tumor immunotherapy, particularly in NK cell therapy (**Table 2**).

Core concept: Why develop CAR-NK and iPSC-NK?

Traditional NK cell therapies (such as using NK92 cell lines or primary NK cells) face several major challenges:

1. Insufficient tumor targeting: NK cells recognize target cells through intrinsic receptors, but tumor cells often evade detection by downregulating MHC-I molecules (“losing self”).
2. Limited cell availability: The number of primary NK cells obtained from donor peripheral blood or umbilical cord blood is limited, and there are individual differences in quality and activity.
3. Allogeneic use barriers: Although the risk of rejection for allogeneic NK cells is lower, there is still a risk of host versus graft reactions, and cells cannot survive long-term in the recipient’s body.

The CAR-NK and iPSC-NK technologies were developed specifically to address these issues.

**CAR-NK Cell Therapy**

CAR-NK, or Chimeric Antigen Receptor NK Cells, operates on principles similar to CAR-T: through genetic engineering, a CAR structure that can specifically recognize tumor antigens is introduced into NK cells, allowing them to bypass their inherent recognition mechanisms and directly, precisely attack tumor cells.

CAR Structure (with a typical example): Extracellular Domain: Single chain variable fragment, used for specifically recognizing tumor-associated antigens (such as CD19, BCMA, HER2, etc.). Hinge Region: Provides flexibility and spatial extension. Transmembrane Domain: Anchors the receptor in the cell membrane. Intracellular Signaling Domain: This is one of the key differences between CAR-NK and CAR-T. In addition to CD3ζ (which provides the primary signal), NK cell-specific co-stimulatory signaling domains are often integrated, such as 2B4 (CD244), DNAM-1 (CD226), or DAP10, DAP12. These signals can more effectively couple with the activation signals of NK cells, enhancing their toxicity and cytokine secretion.

Unique Advantages of CAR-NK Compared to CAR-T:

1. Exceptional Safety:
  Significantly lower risk of cytokine release syndrome and neurotoxicity: The cytokine profile secreted by NK cells (such as IFN-γ, GM-CSF) differs from that of T cells (such as IL-6, IL-1), making it less likely to trigger severe CRS and ICANS.
  No risk of graft-versus-host disease: Allogeneic NK cells do not attack the host’s normal tissues as they do not express T cell receptors. This facilitates the development of “off-the-shelf” products.
2. Multiple Killing Mechanisms:
  CAR-dependent Killing: Target cells are activated and killed through CAR recognition of antigens. Innate Receptor-dependent Killing: Even if tumor antigen downregulation or mutations occur that lead to CAR unrecognition, NK cells can still eliminate tumors through their natural activating receptors (such as NKG2D, DNAM-1, NCRs) and ADCC effects (via endogenous CD16), effectively preventing tumor escape.
3. “Off-the-shelf” Potential: Due to high safety, large-scale preparation, quality control, and cryopreservation from healthy donors or iPSCs can be done in advance, making them readily available for patients in need, significantly reducing costs and time.

**Clinical Trial Progress:**

The most notable case is the targeted CD19 CAR-NK therapy at MD Anderson Cancer Center. This therapy utilizes NK cells derived from umbilical cord blood, with the CAR structure incorporating CD3ζ and 2B4 co-stimulatory domains.

In I/II clinical trials for relapsed/refractory B-cell lymphoma and chronic lymphocytic leukemia, a remission rate of up to 73% was observed, with no severe CRS, ICANS, or GVHD detected. This marks a milestone in the CAR-NK field.

Currently, CAR-NK clinical trials targeting solid tumors (such as HER2, EGFR, MUC1, etc.) and hematologic malignancies (such as BCMA, CD33, etc.) are being widely conducted globally.

**iPSC-induced NK cells**

iPSC, or induced pluripotent stem cells, are stem cells with multidirectional differentiation potential obtained by reprogramming somatic cells (such as skin fibroblasts or peripheral blood cells).

Technical process:

1. Establishing a master cell bank: Create an iPSC cell line with unlimited proliferation capacity from a healthy donor or a specially designed donor cell. This cell line undergoes strict quality control to ensure its safety and stability.
2. Directed differentiation: By simulating the *in vivo* developmental process, specific cytokines and growth factors are sequentially added to the culture system to differentiate iPSCs into hematopoietic progenitor cells, which are then further differentiated into functional NK cells.
3. Genetic engineering: Gene editing (such as CRISPR/Cas9) can be performed at the iPSC stage to introduce CAR, high-affinity CD16, cytokines, etc., resulting in “engineered” NK cells that carry these modified characteristics in all progeny NK cells.
4. Amplification and storage: The differentiated NK cells can be massively amplified and made into “off-the-shelf” frozen products.

**Disruptive advantages of iPSC-NK:**

1. Infinite, renewable cell source: A single iPSC master cell bank can differentiate into tens of thousands or even millions of doses of NK cells, fundamentally solving the bottleneck of cell sourcing.
2. Highly homogenized products: Compared to the individual and batch variabilities present in traditional primary NK cells, iPSC-NK products are highly uniform and quality controllable, ensuring the stability and reproducibility of clinical trials and therapeutic effects.
3. Perfect engineering platform: Genetic modification at the iPSC stage is highly efficient and ensures that all differentiated NK cells carry the same genetic modifications. This makes the bulk production of “enhanced” NK cells possible, such as:

Expressing CAR targeting specific tumors. Knocking out the CD38 gene to avoid mis-targeting by anti-CD38 antibodies during the treatment of multiple myeloma. Expressing high-affinity, non-cleavable CD16 (hnCD16) to significantly enhance ADCC effects. Expressing cytokines (such as IL-15) to promote their survival and persistence *in vivo*.

**Stronger Together: CAR-iPSC-NK**

The most promising direction is to combine both, that is, to differentiate NK cells expressing specific CARs from genetically engineered iPSC cell lines. Process: iPSC → Gene Editing (insert CAR, etc.) → Establish engineered master cell bank →Directed differentiation into NK cells → CAR-iPSC-NK products. This strategy enables the large-scale production of standardized NK cell products that are “off-the-shelf,” highly targeted, and functionally enhanced, representing the future direction of industrialized cell therapy (**Table 2**).

Companies and products in clinical trials:

Fate Therapeutics: A leader in this field. Its flagship product is an iPSC-derived CAR-NK cell that targets CD19 and expresses hnCD16 and IL-15/IL-15 receptor fusion protein (Ghobadi et al., 2025) (https://clinicaltrials.gov/search?intr=FT596). The company also has a pipeline of various products targeting multiple myeloma (FT576 targeting BCMA, Allogenic CAR NK cells with BCMA expression, https://clinicaltrials.gov/search?intr=FT576), all of which have entered clinical stages. Other companies, such as Century Therapeutics (https://www.centurytx.com/) and Wugen (https://wugen.com/), are also actively engaging in this field.

CAR-NK and iPSC-NK technologies are reshaping the landscape of cellular immunotherapy for cancer. They combine the inherent safety and multiple killing mechanisms of NK cells with the precise targeting ability of CAR technology and the unlimited expansion potential of iPSC technology. Although challenges such as *in vivo* persistence and solid tumor infiltration remain, current clinical trial data have demonstrated their immense therapeutic potential and “off-the-shelf” industrialization prospects, offering a new and powerful treatment option for a broader range of patients.

#### 4.3.2 Strategies for enhancing NK cell specificity and *In Vivo* persistence

Several genetic modifications can enhance NK cell specificity and *in vivo* persistence, improving their efficacy against HPV-related tumors. TGF-β receptor knockout and enhanced CD16 expression are two such strategies.

TGF-β is an immunosuppressive cytokine that is often upregulated in the tumor microenvironment, inhibiting NK cell activity and promoting tumor progression (Viel et al., 2016; Shaim et al., 2021; Thangaraj et al., 2024). Genetic modification of NK cells to knockout the TGF-β receptor (TGF-βR) can enhance their resistance to immunosuppression. CRISPR-mediated TGF-βR knockout has been used to generate NK cells that are more resistant to TGF-β-mediated inhibition, resulting in improved antitumor activity in preclinical models (Elmas et al., 2022; Overcoming TGFβ and activin A suppression boosts NK cell antitumor function, 2025).

CD16 (FcγRIIIa) is an Fc receptor expressed on NK cells that mediates ADCC. Enhancement of CD16 expression on NK cells can improve their ability to kill tumor cells coated with therapeutic antibodies, such as cetuximab, which targets EGFR-overexpressing HPV+ cancers (Veluchamy et al., 2017; Baysal et al., 2020). Increased CD16 expression improves ADCC against HPV+ tumors (Baysal et al., 2020).

Limitations of these strategies include potential off-target effects and manufacturing hurdles. Off-target effects can occur when CRISPR-mediated gene editing leads to unintended modifications in the genome, potentially causing adverse effects. Manufacturing hurdles include the challenges of generating large numbers of genetically modified NK cells with high purity and consistency.

### 4.4 Combination therapies and bispecific approaches

#### 4.4.1 Integration with immune checkpoint inhibitors and monoclonal antibodies

Combination therapies integrating NK cells with other immunotherapies or conventional treatments offer synergistic strategies for enhancing antitumor responses. Immune checkpoint inhibitors (ICIs) and monoclonal antibodies, such as cetuximab, are often used in combination with NK cell-based therapies (Baysal et al., 2020).

ICIs, such as anti-PD-1 and anti-PD-L1 antibodies, block inhibitory signaling pathways that suppress T cell and NK cell function. Checkpoint inhibitors (e.g., anti-PD-1) restore NK cell function in HPV-related tumors (Karami et al., 2023). Blocking these pathways can restore NK cell activity and enhance antitumor immunity. In HPV-related tumors, ICIs can reverse T cell exhaustion and promote NK cell-mediated tumor cell killing (Allouch et al., 2020; Aghbash et al., 2023; Li et al., 2024; Zhen, 2025).

Cetuximab is a monoclonal antibody that targets the epidermal growth factor receptor (EGFR), which is often overexpressed in HPV+ cancers (Zhang et al., 2022). Cetuximab engages NK cells via CD16 to target EGFR-overexpressing HPV+ cancers (Johnson et al., 2020; Alsahafi et al., 2021). Cetuximab induces natural killer cell cytotoxicity in head and neck squamous cell carcinoma cell lines (Baysal et al., 2020). Clinical trial outcomes and resistance mechanisms in cetuximab-based therapies have been studied (Economopoulou and Psyrri, 2019; Gillison et al., 2019; Mehanna et al., 2019).

Clinical trials evaluating the combination of ICIs and cetuximab with NK cell therapies are ongoing. Preliminary results suggest that this combination can improve clinical outcomes in patients with HPV-related malignancies.

#### 4.4.2 Synergistic effects with vaccination or conventional treatments

Therapeutic HPV vaccines and conventional treatments, such as chemotherapy and radiotherapy, can synergize with NK cell-based therapies to enhance antitumor responses.

Therapeutic HPV vaccines prime NK cell responses. Therapeutic HPV vaccines (e.g., E6/E7-targeting vaccines) prime NK cell responses, leading to improved tumor control. These vaccines stimulate T cell responses against HPV antigens, which in turn activate NK cells (1114, Li et al., 2024).

NK cell activation occurs in the context of chemotherapy/radiotherapy-induced immunogenic cell death. Chemotherapy and radiotherapy can induce immunogenic cell death (ICD) in tumor cells, leading to the release of damage-associated molecular patterns (DAMPs) that activate NK cells and other immune cells (Rapoport and Anderson, 2019; Fang et al., 2025). Combination regimens of chemotherapy or radiotherapy with NK cell therapies have shown promising results in recent studies.

## 5 Clinical applications and progress

### 5.1 Overview of clinical trials involving NK cell-based therapies in HPV-related tumors

#### 5.1.1 Data from CIN and cervical cancer studies

Clinical trials involving NK cell-based therapies for CIN and cervical cancer are exploring various strategies to harness the cytotoxic potential of NK cells. One such approach involves the use of Poly-gamma-glutamic acid (γ-PGA), an oral medication, to stimulate the immune system. A multicenter, randomized, double-blind, phase II trial investigated the short-term efficacy and safety of γ-PGA in women with CIN 1 (Cho et al., 2019). The results showed that γ-PGA significantly improved the histologic regression rate of CIN 1 at 12 weeks compared to the control group (42.4% vs. 27.1%, p = 0.018) (Cho et al., 2019). Furthermore, HPV clearance was significantly higher in the γ-PGA group among patients infected with high-risk HPV (43.5% vs. 26.7%, p = 0.026) (Cho et al., 2019). However, the study found no significant difference in the change of NK cell activity, MHC class II CD8 count, and CD56 count between the two groups (Cho et al., 2019).

Adoptive transfer of allogeneic NK cells is another area of active investigation. Veluchamy et al. explored the cytotoxic efficacy of activated allogeneic NK cells from peripheral blood (PBNK) and umbilical cord blood (UCB-NK) on cervical cancer cell lines. The study revealed that UCB-NK cells achieved superior cytotoxicity compared to activated PBNK cells alone (Veluchamy et al., 2017). Both PBNK- and UCB-NK-mediated cytotoxic activity was dependent on the NK-activating receptors NKG2D and DNAM-1 (Veluchamy et al., 2017). Notably, while PBNK’s cytotoxic activity was inversely correlated with HLA-ABC levels, UCB-NK cytotoxicity was entirely independent of HLA-ABC expression. This suggests that UCB-NK cells might serve as a generally applicable treatment for cervical cancer, enabled by HLA-, histology- and HPV-independent killing mechanisms (Veluchamy et al., 2017).

Another study used single-cell RNA sequencing (scRNA-Seq) to investigate the impact of hapten-enhanced intratumoral chemotherapy (HEIC) on precancerous cervical lesions with HPV (Liu et al., 2023). The study found that intratumoral injection of chemotherapy drug plus hapten induces an acute immune response and awakens immune cells to prevent the abnormal proliferation of the precancerous cells (Liu et al., 2023).

These studies highlight the potential of NK cell-based therapies in the treatment of CIN and cervical cancer. Further clinical trials are needed to optimize these approaches and evaluate their long-term efficacy and safety.

#### 5.1.2 NK cell therapies in HPV-associated head and neck cancers

NK cell therapies are also being explored in HPV-associated HNSCC, a malignancy with increasing incidence (Koneva et al., 2018). Mandal et al. analyzed transcriptome data from HNSCC tumors and found that both HPV (+) and HPV (-) HNSCC tumors are among the most highly immune-infiltrated cancer types (Mandal et al., 2016). Strikingly, HNSCC had the highest median Treg/CD8^+^ T cell ratio and the highest levels of CD56^dim^ NK cell infiltration in their pan-cancer analysis (Mandal et al., 2016). Furthermore, CD8^+^ T cell infiltration and CD56^dim^ NK cell infiltration each correlated with superior survival in HNSCC (Mandal et al., 2016).

Westrich et al. demonstrated that the chemokine CXCL14 is significantly downregulated by the HPV oncoprotein E7 during cancer progression (Westrich et al., 2019). Restoration of CXCL14 expression in HPV-positive HNC cells dramatically suppresses tumor growth and increases survival through an immune-dependent mechanism in mice. CD8^+^ T cells are required for CXCL14-mediated tumor suppression, and CXCL14 expression restores MHC-I expression on HPV-positive HNC cells (Westrich et al., 2019).

Baysal et al. investigated cetuximab-induced natural killer cell cytotoxicity in head and neck squamous cell carcinoma cell lines (Baysal et al., 2020). The study found that intrinsic cetuximab-resistant cell lines showed an increased ADCC induction, whereas exposure to hypoxia reduced ADCC. EGFR internalisation following cetuximab treatment was positively correlated with ADCC. These findings suggest that resistance against cetuximab can be overcome by NK cell-based immune reactions (Baysal et al., 2020).

Lin et al. (2024) used scRNA-seq to explore the immune heterogeneity of NK cells in HPV-positive HNSCC (Lin et al., 2024). They identified a specific type of tissue-resident NK cell (trNK-0) that was involved in the anti-tumor and immunotherapy response in HPV-positive HNSCC.

These studies suggest that NK cell therapies may be a promising approach for treating HPV-associated HNSCC. However, challenges specific to head and neck tumors, such as accessibility and tumor heterogeneity, need to be addressed.

### 5.2 Autologous, Allogeneic, and engineered NK cell approaches

#### 5.2.1 Comparative efficacy of different NK cell sources

The choice of NK cell source, whether autologous or allogeneic, is a critical consideration in the design of NK cell-based therapies (**Figure 6B**). Autologous NK cells, derived from the patient’s own blood, offer the advantage of minimal risk of graft-versus-host disease (GVHD) and immunological rejection (Veluchamy et al., 2017). However, autologous NK cells may exhibit impaired function due to tumor-induced immunosuppression or prior treatment (Zhang et al., 2019). Furthermore, obtaining a sufficient number of autologous NK cells for therapeutic purposes can be challenging, particularly in patients with compromised immune systems (Veluchamy et al., 2017) (**Table 2**).

Allogeneic NK cells, sourced from a healthy donor, provide a potentially more potent and readily available source of cytotoxic cells (Veluchamy et al., 2017). Allogeneic NK cells can mediate anti-tumor effects through HLA mismatch, leading to enhanced activation and cytotoxicity against tumor cells that have downregulated HLA expression (Veluchamy et al., 2017). However, allogeneic NK cell therapy carries the risk of GVHD, although NK cell-mediated GVHD is generally less severe than that associated with T cell transplantation (Veluchamy et al., 2017; Chan et al., 2018).

Umbilical cord blood (UCB) is an alternative source of allogeneic NK cells that offers several advantages. UCB-NK cells are relatively immature and exhibit a broader repertoire of activating receptors, making them capable of targeting a wider range of tumor cells. UCB-NK cells also have a lower risk of GVHD compared to adult peripheral blood NK cells (Veluchamy et al., 2017). Furthermore, UCB is a readily available and well-characterized source of NK cells, facilitating the development of “off-the-shelf” NK cell products (de Molla et al., 2023).

Induced pluripotent stem cells (iPSCs) represent another promising source of NK cells for cancer immunotherapy. iPSC-derived NK cells can be generated in large quantities and genetically modified to enhance their anti-tumor activity (Veluchamy et al., 2017). iPSC-NK cells also have a low risk of GVHD, making them an attractive option for allogeneic cell therapy (Hsu et al., 2021; Zong and Wang, 2025) (**Table 2**).

#### 5.2.2 Implementation of genetic modifications in clinical settings

Genetic engineering holds great promise for enhancing the efficacy and safety of NK cell therapies. Chimeric antigen receptors (CARs) are synthetic receptors that redirect NK cell cytotoxicity towards tumor cells expressing specific antigens (Veluchamy et al., 2017). CAR-NK cells have shown promising preclinical activity against various cancers, including hematological malignancies and solid tumors (Xie et al., 2020; Peng et al., 2024). Several clinical trials are currently evaluating the safety and efficacy of CAR-NK cell therapy in patients with cancer (Peng et al., 2024) (**Table 2**).

Another approach to enhance NK cell function is to knockout TGF-β receptor. Mukherjee et al. reported that TriCurin, a synergistic formulation of curcumin, resveratrol, and epicatechin gallate, repolarizes tumor-associated macrophages and triggers an immune response to cause suppression of HPV+ tumors (Mukherjee et al., 2018). TriCurin injection repolarized M2-type tumor-associated macrophages (TAM) into M1-type TAM in HPV+ tumors (Mukherjee et al., 2018). Neutralizing IL12 signaling with an IL12 antibody abrogated TriCurin-induced intra-tumor entry of activated NK cells and Cytotoxic T lymphocytes (CTL), thereby confirming that IL12 triggers recruitment of NK cells and CTL (Mukherjee et al., 2018).

Enhancing CD16 expression on NK cells is another strategy to improve their anti-tumor activity. CD16 is an Fc receptor that mediates ADCC (Baysal et al., 2020). Overexpression of CD16 on NK cells can enhance their ability to kill tumor cells coated with therapeutic antibodies, such as cetuximab (Baysal et al., 2020).

Furthermore, genetic modifications can be used to improve NK cell persistence and resistance to immunosuppressive environments. For example, knocking out genes encoding inhibitory receptors, such as TIGIT or KLRG1, can enhance NK cell activation and cytotoxicity (Mandal et al., 2016).

The implementation of genetic modifications in clinical settings requires careful consideration of safety and regulatory issues. However, the potential benefits of genetically engineered NK cells in cancer immunotherapy are substantial.

### 5.3 Combination therapeutic strategies and their clinical outcomes

#### 5.3.1 NK cells combined with chemoradiotherapy and immunomodulatory agents

Combining NK cell-based therapies with conventional treatments like chemotherapy and radiotherapy, as well as immunomodulatory agents, represents a promising strategy to enhance anti-tumor efficacy. Chemotherapy and radiotherapy can induce ICD, releasing tumor-associated antigens that stimulate the immune system (Gong et al., 2021). Combining NK cell therapy with chemoradiotherapy can amplify this effect, leading to enhanced tumor cell killing and long-lasting anti-tumor immunity (Gong et al., 2021).

Immunomodulatory agents, such as cytokines and immune checkpoint inhibitors, can further enhance NK cell activity and overcome tumor-induced immunosuppression. Cytokines like IL-2, IL-12, and IL-15 can stimulate NK cell proliferation, activation, and cytotoxicity. Immune checkpoint inhibitors, such as anti-PD-1 and anti-CTLA-4 antibodies, can block inhibitory signals that suppress NK cell function, leading to enhanced anti-tumor activity (Khan et al., 2020; Song et al., 2025).

A phase I/II study of the Aurora Kinase A Inhibitor Alisertib and Pembrolizumab in Refractory, Rb-Deficient HNSCC showed that the combination of alisertib and pembrolizumab was well tolerated and led to prolonged stable disease in some immunotherapy-resistant patients (Gong et al., 2021). In circulating immune cells and plasma, patients with stable disease had markedly higher levels of HLA *de novo* resistance-expressing NK cells than did progressive disease patients who demonstrated a more immunosuppressive and inflammatory profile (Gong et al., 2021).

#### 5.3.2 Case studies and biomarker-based outcome evaluations

Case studies provide valuable insights into the clinical impact of NK cell therapies in real-world scenarios (**Table 3**). For example, patients who demonstrate exceptional responses to NK cell therapy can provide clues about the mechanisms of action and predictive biomarkers.

**Table 3:** NK cells application in clinical trials.

NCT number	Study title	Study status	Conditions	Interventions	Sponsor	Phases	Study type
NCT02849340	Combination of Cryosurgey and NK Immunotherapy for Recurrent Cervical Cancer	Completed	Recurrent Cervical Cancer	Device: Cryosurgery Biological: NK immunotherapy	Fuda Cancer Hospital, Guangzhou	Phase1 Phase2	Interventional
NCT02507154	Reactivating NK Cells in Treating Refractory Head and Neck Cancer	Unknown	Nasopharyngeal Cancer Head and Neck Squamous Cell Carcinoma	Drug: Cetuximab + NK cells	National University Hospital, Singapore	Phase1 Phase2	Interventional
NCT02843581	Combination of Cryosurgery and NK Immunotherapy for Advanced Esophageal Cancer	Completed	Metastatic Esophageal Cancer	Device: Cryosurgery Biological: NK immunotherapy	Fuda Cancer Hospital, Guangzhou	Phase1 Phase2	Interventional
NCT04290546	CIML NK Cell in Head & Neck Cancer	Completed	Squamous Cell Carcinoma of the Head and Neck Recurrent Head and Neck Squamous Cell Carcinoma	Drug: Interleukin-15 Superagonist (N-803) Biological: CIML NK cell Infusion Drug: Ipilimumab Drug: Cetuximab	Dana-Farber Cancer Institute	Phase1	Interventional
NCT02849379	Combination of Cryosurgey and NK Immunotherapy for Recurrent Tongue Cancer	Completed	Recurrent Tongue Cancer	Device: Cryosurgery Biological: NK immunotherapy	Fuda Cancer Hospital, Guangzhou	Phase1 Phase2	Interventional
NCT02849327	Combination of Cryosurgey and NK Immunotherapy for Recurrent Pharyngeal Cancer	Completed	Recurrent Pharyngeal Cancer	Device: Cryosurgery Biological: NK immunotherapy	Fuda Cancer Hospital, Guangzhou	Phase1 Phase2	Interventional
NCT02849314	Combination of Cryosurgey and NK Immunotherapy for Recurrent Laryngeal Cancer	Completed	Recurrent Laryngeal Cancer	Device: Cryosurgery Biological: NK immunotherapy	Fuda Cancer Hospital, Guangzhou	Phase1 Phase2	Interventional
NCT04847466	Immunotherapy Combination: Irradiated PD-L1 CAR-NK Cells Plus Pembrolizumab Plus N-803 for Subjects With Recurrent Metastatic Gastric Head and Neck Cancer	Recruiting	Gastroesophageal Junction (GEJ) Cancers Advanced HNSCC	Drug: N-803 Drug: Pembrolizumab Biological: PD-L1 t-haNK	National Cancer Institute (NCI)	Phase2	Interventional
NCT04106167	Long-term, Non-interventional, Observational Study Following Treatment With Fate Therapeutics FT500 Cellular Immunotherapy	Terminated	Advanced Solid Tumor Lymphoma Gastric Cancer Colorectal Cancer Head and Neck Cancer Squamous Cell Carcinoma EGFR Positive Solid Tumor HER2-positive Breast Cancer Hepatocellular Carcinoma Small-cell Lung Cancer Renal Cell Carcinoma Pancreas Cancer Melanoma NSCLC Urothelial Carcinoma Cervical Cancer Microsatellite Instability Merkel Cell Carcinoma	Genetic: Allogeneic natural killer (NK) cell	Fate Therapeutics		Observational
NCT03420963	Donor Natural Killer Cells, Cyclophosphamide, and Etoposide in Treating Children and Young Adults With Relapsed or Refractory Solid Tumors	Recruiting	Recurrent Cutaneous Melanoma Recurrent Lip and Oral Cavity Carcinoma Recurrent Malignant Endocrine Neoplasm Recurrent Malignant Female Reproductive System Neoplasm Recurrent Malignant Male Reproductive System Neoplasm Recurrent Malignant Mesothelioma Recurrent Malignant Neoplasm of Multiple Primary Sites Recurrent Malignant Oral Neoplasm Recurrent Malignant Pharyngeal Neoplasm Recurrent Malignant Skin Neoplasm Recurrent Malignant Soft Tissue Neoplasm Recurrent Malignant Solid Neoplasm Recurrent Malignant Thyroid Gland Neoplasm Recurrent Malignant Urinary System Neoplasm Refractory Cutaneous Melanoma|Refractory Malignant Bone Neoplasm|Refractory Malignant Endocrine Neoplasm Refractory Malignant Female Reproductive System Neoplasm Refractory Malignant Male Reproductive System Neoplasm Refractory Malignant Mesothelioma Refractory Malignant Neoplasm of Multiple Primary Sites Refractory Malignant Oral Neoplasm Refractory Malignant Pharyngeal Neoplasm Refractory Malignant Skin Neoplasm Refractory Malignant Soft Tissue Neoplasm Refractory Malignant Solid Neoplasm Refractory Malignant Thyroid Gland Neoplasm Refractory Malignant Urinary System Neoplasm	Biological: Cord Blood-derived Expanded Allogeneic Natural Killer Cells Drug: Cyclophosphamide Drug: Etoposide	M.D. Anderson Cancer Center	Phase1	Interventional

Biomarker analyses that correlate treatment success with baseline characteristics can help to identify patients who are most likely to benefit from NK cell therapy. Potential biomarkers include NK cell activation status, ligand expression levels on tumor cells, and circulating cytokine profiles.

Li et al. found that lower CD4^+^ T cells were associated with a higher risk of HPV, high-risk HPV, HPV18 and HPV52 infections (Li et al., 2023). Lower T-cell and CD8^+^ T-cell counts, as well as a higher NK cell count, are unfavorable factors for natural HPV clearance.

Mastrolonardo et al. employed response-adaptive surgical timing to identify responders to immunotherapy and enhance their response in HNSCC) (Mastrolonardo et al., 2025). A pretreatment NK cell signature, PD-L1 status, and IFN-γ expression in the HPV- cohort correlated with response (Ting et al., 1995).

Wichmann et al. demonstrated in HPV-driven HNSCC a deviating distribution of HLA antigens and haplotypes and their relevance to outcome (Wichmann et al., 2023). Together with alcohol consumption, tobacco smoking, T category, and extranodal extension of locoregional metastases and treatment applied, eight HLA traits allow for predicting progression-free and tumor-specific survival.

Soleymaninejadian et al. reviewed the recent progression in cellular and humoral immunity studies during the progression of HPV-related cancers and describe the role of NK cells in tumor progression and prevention (Soleymaninejadian et al., 2022).

Personalized medicine approaches that tailor NK cell therapy to individual patients based on predictive biomarkers are essential to optimize treatment outcomes.

## 6 Challenges and future directions

### 6.1 Limitations and obstacles in NK cell therapy

#### 6.1.1 *In Vivo* persistence, trafficking, and expansion challenges

One of the primary limitations of NK cell therapy is the relatively short *in vivo* lifespan of infused NK cells, which restricts their ability to effectively target and eliminate tumor cells over an extended period (Westrich et al., 2019; William et al., 2021). This is particularly relevant in the context of HPV-related tumors, where sustained immune surveillance is crucial for preventing recurrence. The limited persistence of NK cells post-infusion can be attributed to several factors, including the lack of survival signals in the recipient, immune-mediated rejection, and the absence of continuous stimulation needed to maintain their cytotoxic activity (Soleymaninejadian et al., 2022).

Effective trafficking of NK cells to tumor sites, especially in solid tumors like those associated with HPV, presents another significant challenge. The tumor microenvironment (TME) often contains physical barriers and lacks the necessary chemotactic signals to attract NK cells (Westrich et al., 2019). Solid tumors can exhibit reduced expression of chemokines like CXCL9, which are important for recruiting immune cells, including NK cells (William et al., 2021). Additionally, the tumor vasculature can be abnormal, hindering the extravasation of NK cells into the tumor parenchyma (Cicchini et al., 2016).

Various strategies have been explored to improve the *in vivo* persistence and expansion of NK cells. Cytokine support, such as IL-15 and IL-2, can enhance NK cell survival and proliferation (Battella et al., 2016; Amador-Molina et al., 2019). Modifying cell culture conditions prior to infusion, including pre-activation with cytokines or co-stimulatory molecules, can also improve their functional capacity and longevity (Mukherjee et al., 2018). Furthermore, genetic engineering approaches, like introducing genes that promote survival or enhance cytokine responsiveness, are being investigated to generate NK cells with improved persistence (Luetke-Eversloh et al., 2013; Wagner et al., 2016).

#### 6.1.2 Immunosuppressive tumor microenvironment and immune escape mechanisms

HPV-related tumors often exhibit an immunosuppressive microenvironment that significantly impairs NK cell function. Factors such as TGF-β and IL-10 are commonly present in these tumor environments, suppressing NK cell activity and promoting immune evasion (Mukherjee et al., 2018; Khan et al., 2020; Song et al., 2025). TGF-β, for example, inhibits NK cell cytotoxicity and IFN-γ production, while IL-10 promotes the differentiation of Tregs, which further dampen the immune response (Mukherjee et al., 2018).

HPV-modulated tumor cells employ various mechanisms to evade NK cell recognition and clearance. Downregulation of MHC class I molecules is a common strategy, reducing the presentation of tumor-associated antigens to T cells, and can also affect NK cell activation, as NK cells are regulated by the balance of activating and inhibitory signals, and the absence of MHC-I can prevent inhibitory signals (Gutiérrez-Hoya et al., 2019; Westrich et al., 2019). Additionally, HPV oncoproteins, such as E6 and E7, can directly interfere with immune signaling pathways and promote the expression of inhibitory ligands on tumor cells, such as PD-L1 (Cicchini et al., 2016).

Tumors can develop resistance to NK cell responses through multiple mechanisms. Alterations in the expression of NK cell activating ligands, such as NKG2D ligands, can reduce NK cell recognition and cytotoxicity (Veluchamy et al., 2017; Li and Liu, 2022). Some tumors also secrete factors that directly inhibit NK cell function or promote NK cell exhaustion, characterized by reduced cytokine production and cytotoxicity (Zhang et al., 2019). Furthermore, the upregulation of immune checkpoint molecules, like TIGIT and KLRG1, on NK cells can further suppress their activity, allowing tumors to escape immune surveillance (Mandal et al., 2016).

### 6.2 Strategies to overcome clinical and biological barriers

#### 6.2.1 Technological advances in genetic engineering and nanoparticle delivery

Recent advancements in genetic engineering techniques offer promising avenues for enhancing NK cell functionality. CRISPR/Cas9 technology allows for precise modification of NK cell receptors, improving their cytotoxic capabilities and specificity (Mandal et al., 2016; William et al., 2021). For example, disrupting inhibitory receptors like TIGIT or KIRs can enhance NK cell activation and anti-tumor responses (Mandal et al., 2016). Moreover, CAR-NK cells, engineered to express chimeric antigen receptors (CARs), can be designed to target specific tumor-associated antigens, enhancing their specificity and cytotoxic activity against HPV-related tumors (Luetke-Eversloh et al., 2013; Wagner et al., 2016).

Nanoparticle systems can be employed to deliver cytokines or genetic materials to enhance NK cell proliferation and persistence. These systems can protect their cargo from degradation and deliver them directly to NK cells or the tumor microenvironment. For instance, nanoparticles loaded with IL-15 can stimulate NK cell proliferation and survival, while nanoparticles delivering siRNA targeting immunosuppressive factors can modulate the TME (Amador-Molina et al., 2019).

Several preliminary studies demonstrate the efficacy of these approaches. CAR-NK cells targeting specific tumor antigens have shown promising results in preclinical models, exhibiting enhanced cytotoxicity and tumor control (Luetke-Eversloh et al., 2013; Wagner et al., 2016). Nanoparticle-mediated delivery of cytokines has also demonstrated improved NK cell activation and anti-tumor responses *in vivo* (Amador-Molina et al., 2019). These advancements hold great potential for improving the clinical efficacy of NK cell therapies in HPV-related tumors.

#### 6.2.2 Personalized and precision immunotherapy approaches based on biomarker signatures

Tailoring NK cell therapies based on specific tumor biomarkers presents a promising strategy for improving clinical outcomes in HPV-related cancers. Specific tumor biomarkers can predict which patients are most likely to respond to NK cell therapies. For instance, PD-L1 expression, MHC class I expression, or the presence of specific HPV genotypes can serve as predictive biomarkers (Westrich et al., 2019; Rafei et al., 2021).

Integrating personalized medicine with NK cell therapy could significantly improve clinical outcomes. For example, patients with tumors expressing high levels of inhibitory ligands might benefit from NK cells engineered to resist these inhibitory signals (Mandal et al., 2016). Similarly, patients with specific HLA types might benefit from NK cells selected to match their HLA profile, reducing the risk of NK cell rejection (Veluchamy et al., 2017).

Emerging research findings support the potential of personalized NK cell therapy. Studies have shown that NK cell activity and responsiveness vary significantly among individuals, highlighting the need for personalized approaches. By identifying predictive biomarkers and tailoring NK cell therapies accordingly, it may be possible to achieve greater clinical efficacy and reduce the risk of treatment failure.

### 6.3 Prospective research and translational opportunities

#### 6.3.1 Emerging combination modalities and next-generation NK cell therapies

Combining NK cell therapies with other treatment modalities, such as checkpoint inhibitors or conventional chemotherapy, offers a promising strategy for improving therapeutic outcomes. Checkpoint inhibitors, like anti-PD-1 or anti-CTLA-4 antibodies, can block inhibitory signals and enhance NK cell activity, while chemotherapy can reduce tumor burden and improve NK cell access to tumor cells. The rationale behind such combinations is to create a synergistic effect, where each modality enhances the efficacy of the other, leading to more durable and complete responses.

New strategies, such as bi-specific T-cell engagers (BiTEs) or tumor-targeted NK cells, are in development or early clinical trials. BiTEs are designed to simultaneously bind NK cells and tumor cells, facilitating NK cell-mediated cytotoxicity (William et al., 2021). Tumor-targeted NK cells, engineered to express receptors that specifically recognize tumor-associated antigens, can enhance their specificity and cytotoxic activity against tumor cells (Wagner et al., 2016).

These emerging modalities hold great promise for improving the clinical efficacy of NK cell therapies. By combining NK cell therapy with other treatment modalities or developing novel strategies like BiTEs and tumor-targeted NK cells, it may be possible to overcome the limitations of current therapies and achieve more durable and complete responses in HPV-related tumors (Dao et al., 2022).

#### 6.3.2 Integration of basic mechanistic insights with clinical trial design

Integrating mechanistic research findings into the design of clinical trials is crucial for ensuring that trials are informed by underlying biological principles that affect NK cell efficacy. Understanding the complex interactions between NK cells and the tumor microenvironment can help identify potential targets for therapeutic intervention and optimize trial outcomes (Soleymaninejadian et al., 2022).

Future studies should account for the complexity of NK cell interactions within the HPV-related tumor microenvironment to optimize trial outcomes. This includes considering factors such as NK cell trafficking, activation, and suppression, as well as the impact of other immune cells and stromal components on NK cell function (Cicchini et al., 2016).

Approaches or frameworks for designing future studies include incorporating biomarker analysis to identify patients most likely to respond to NK cell therapy, using preclinical models to evaluate the efficacy of different NK cell therapies and combination strategies, and employing adaptive trial designs to adjust treatment strategies based on early response data. By integrating basic mechanistic insights with clinical trial design, it may be possible to develop more effective and personalized NK cell therapies for HPV-related tumors.

## 7 Conclusions and outlook

### 7.1 Summary of key mechanistic insights into NK cell activation in HPV-related tumor control

**Figure 7:**
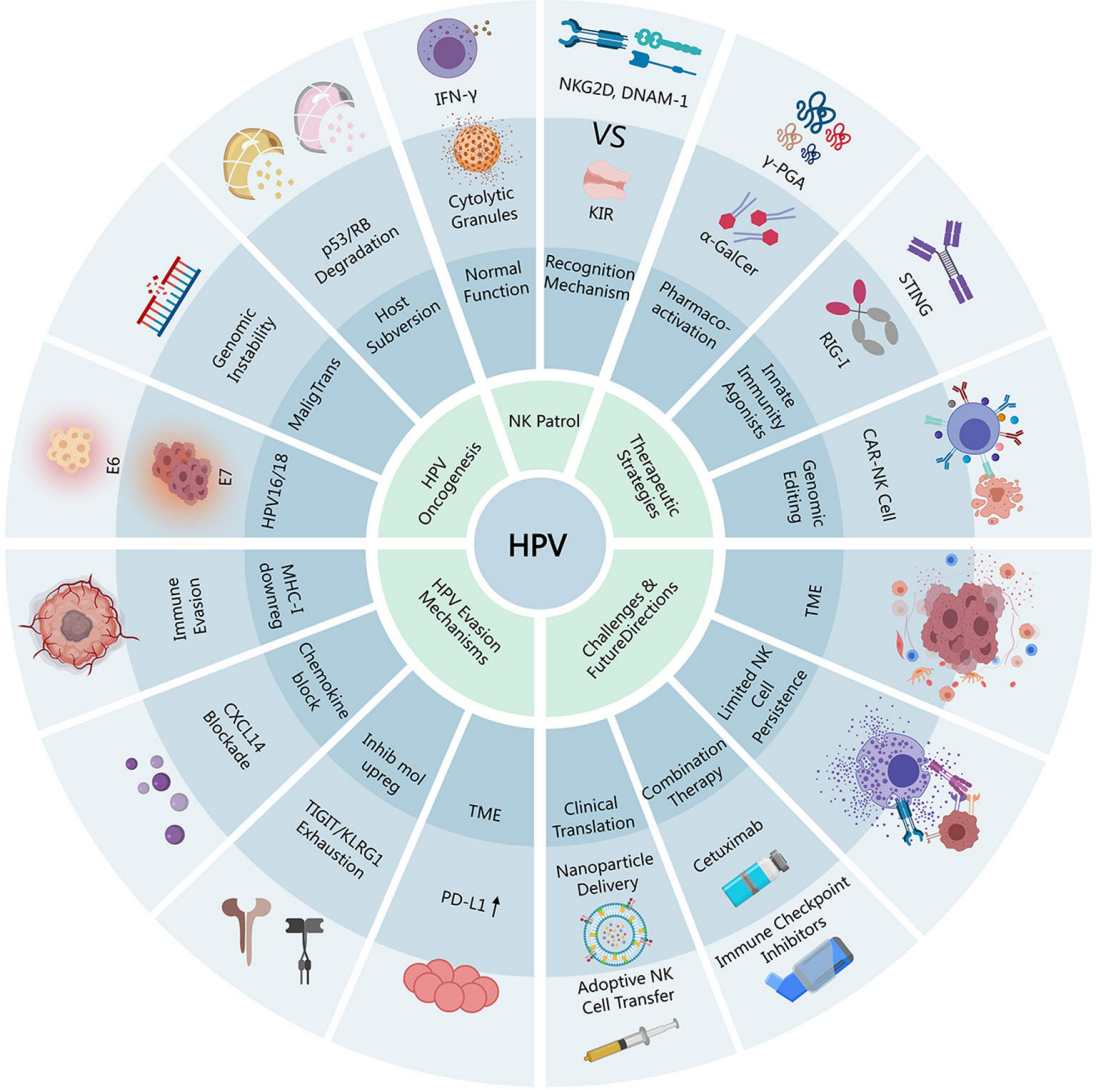
Circular diagram illustrating HPV research topics. Sections include HPV Oncogenesis, Evasion Mechanisms, NK Cell Patrol, Therapeutic Strategies, and Challenges. Subtopics feature genomic instability, Immune evasion, Pharmacologic activation, CAR-NK cell, and therapies like combination therapy, and immune checkpoint inhibitors.

NK cells are crucial components of the innate immune system, providing rapid responses against viral infections and tumors (Soleymaninejadian et al., 2022) (**Figure 7**). Their cytotoxic function involves direct lysis of target cells and secretion of cytokines like IFN-γ, which bridge innate and adaptive immunity (Soleymaninejadian et al., 2022). NK cell activation is governed by a delicate balance between activating and inhibitory receptor signals. Activating receptors, such as NKG2D and DNAM-1, recognize stress-induced ligands on target cells, triggering NK cell effector functions. Inhibitory receptors, including KIRs, NKG2A, TIGIT, and KLRG1, recognize MHC-I molecules, preventing NK cell activation against healthy cells (Zhang et al., 2019) (**Figure 7**).

However, HPV employs several strategies to evade immune surveillance. HPV oncoproteins, particularly E6 and E7, play a central role in impairing NK cell function. One mechanism involves downregulating MHC-I expression, making infected cells less susceptible to T cell recognition but potentially more vulnerable to NK cell-mediated killing (Westrich et al., 2019). Paradoxically, HPV also alters chemokine signaling, such as downregulating CXCL14, which reduces NK cell infiltration into the tumor microenvironment (Cicchini et al., 2016; Westrich et al., 2019). Furthermore, HPV infection can induce the expression of inhibitory receptors like TIGIT and KLRG1 on NK cells, leading to NK cell exhaustion and reduced cytotoxicity (Zhang et al., 2019).

Preclinical and clinical studies have shown a clear correlation between NK cell dysfunction and HPV persistence, as well as tumor progression (Zhang et al., 2019) (**Figure 6A**). Conversely, strategies aimed at activating NK cells, such as cytokine stimulation (IL-15, IL-12), modulation of activating receptors (NKG2D), or genetic engineering (CAR-NK cells), have demonstrated enhanced tumor clearance in HPV-associated malignancies (Girone et al., 2023). For example, TriCurin, a combination of curcumin, resveratrol, and epicatechin gallate, has been shown to repolarize tumor-associated macrophages and trigger an immune response, leading to the suppression of HPV+ tumors (Mukherjee et al., 2018). CXCL14 expression restores MHC-I expression on HPV-positive HNSCC cells and promotes antigen-specific CD8^+^ T-cell responses to suppress HPV-positive HNC (Westrich et al., 2019).

Understanding these intricate mechanisms is crucial for developing effective strategies to restore NK cell function in HPV-associated malignancies. By targeting specific pathways involved in HPV-mediated immune evasion, researchers aim to unleash the full potential of NK cells to control and eliminate HPV-infected cells and tumors.

### 7.2 Translational implications for future immunotherapy strategies

Current clinical approaches to harness NK cell-based therapies in HPV-related cancers are showing promise, particularly in cervical cancer and head and neck cancers (Manjgaladze et al., 2019). Adoptive NK cell transfer, where patients receive expanded and activated NK cells, has demonstrated some success in early clinical trials. CAR-NK cells, engineered to express chimeric antigen receptors targeting tumor-associated antigens, offer enhanced specificity and cytotoxicity (Luetke-Eversloh et al., 2013). Cytokine-boosted NK therapies, using cytokines like IL-2 or IL-15 to enhance NK cell activity, are also being explored (Mukherjee et al., 2018) (**Figure 7**).

Combination strategies hold great potential for improving the efficacy of NK cell therapies. Integrating NK cell therapies with immune checkpoint inhibitors (e.g., anti-PD-1/PD-L1 antibodies), monoclonal antibodies (e.g., cetuximab), or conventional treatments (chemoradiotherapy) can create synergistic effects. For instance, combining cetuximab with NK cells enhances ADCC in head and neck squamous cell carcinoma cell lines (Baysal et al., 2020). Dual anti-PD-(L)1/TGF-β inhibitors can also reinvigorate effector activity of NK cells (Viel et al., 2016; Hsu et al., 2018; Cichocki et al., 2020; Shaim et al., 2021; Shokouhifar et al., 2021; Thangaraj et al., 2024).

Despite these advancements, several challenges remain in translating NK cell therapies to the clinic. NK cell persistence after adoptive transfer is often limited, and the immunosuppressive tumor microenvironment can dampen NK cell activity (Cicchini et al., 2016; Westrich et al., 2019). Moreover, patient-specific variability in immune response can lead to inconsistent outcomes. Overcoming these hurdles requires innovative strategies to improve NK cell survival, enhance tumor infiltration, and counteract immunosuppressive factors.

Personalized immunotherapy approaches, guided by biomarkers, are essential to optimize NK cell activation and patient stratification. Identifying predictive biomarkers that correlate with response to NK cell therapy can help select patients most likely to benefit and tailor treatment strategies accordingly. For example, pretreatment NK cell signatures and IFN-γ expression in HPV-negative HNSCC correlate with response to immunotherapy. In HPV-positive HNSCC, B-cell and cancer-associated fibroblast signatures predict response or nonresponse. CD4^+^ T cells were important determinants of HPV infection. T cells, NK cells, and CD8^+^ T cells can serve as potential biomarkers for predicting natural HPV clearance.

### 7.3 Future perspectives on integrating NK cell-based therapies in the clinical management of HPV-associated cancers

Emerging technologies are poised to revolutionize NK cell-based therapies. Next-generation NK cell engineering, such as using iPSC-derived NK cells or CRISPR-based modifications, offers the potential for “off-the-shelf” NK cell products with enhanced functionality and scalability (Luetke-Eversloh et al., 2013). Novel delivery systems, including nanoparticles and STING agonists, can improve NK cell specificity and durability by delivering targeted payloads to the tumor microenvironment (Girone et al., 2023).

Future clinical trials should focus on optimized dosing regimens, combination strategies, and long-term efficacy and safety assessments. Adaptive trial designs, where treatment is adjusted based on early response, can help maximize patient benefit. Evaluating minimal residual disease (MRD) and long-term immune surveillance is crucial to understanding the durability of NK cell-based therapies. Response-adaptive surgical timing enhances treatment response.

The potential of NK cell activation extends beyond treatment to prevention. In prophylactic settings, NK cell-based strategies could be used to prevent the progression of high-grade cervical lesions to invasive cancer. This approach could involve boosting NK cell activity through vaccination or immunomodulatory agents in individuals at high risk of HPV-related malignancies. γ-PGA showed a short-term therapeutic effect on CIN 1 and high-risk HPV infection (Cho et al., 2019). Vaccination with HPV-18 E1 protein plus α-galactosyl-ceramide induces CD8^+^ cytotoxic response and impairs the growth of E1-expressing tumors (Amador-Molina et al., 2019).

Integrating NK cell therapies into existing HPV cancer management protocols requires a multifaceted approach. This includes early intervention strategies for precancerous lesions, adjuvant therapies to prevent recurrence after standard treatment, and personalized approaches based on individual patient characteristics and tumor biology.

Ultimately, NK cell-based immunotherapy holds immense promise as a transformative approach in HPV-associated malignancies. By leveraging mechanistic insights, embracing technological advancements, and conducting rigorous clinical trials, researchers and clinicians can unlock the full potential of NK cells to conquer HPV-related cancers (**Table 3**). A deeper understanding of the immune landscape in HPV-related cancers will be required for further progress in HPV+ immuno-oncology (Mandal et al., 2016). Targeting modulators of Tregs (e.g., CTLA-4, GITR, ICOS, IDO, and VEGFA) and NK cells (e.g., KIR, TIGIT, and 4-1BB) as adjuncts to anti-PD-1 in the treatment of HPV-related cancers may be a novel rationale (Mandal et al., 2016).

## Glossary

ADCC: Antibody Dependent Cell Cytotoxicity
APCs: Antigen-presenting cells
BiTEs: Bi-specific T-cell engagers
CAR-NK: Chimeric antigen receptor-NK
CIN: Intraepithelial neoplasia
CTLs: Cytotoxic T lymphocytes
DAMPs: Damage-associated molecular patterns
DCs: Dendritic cells
DNAM-1: DNAX accessory molecule-1
EGFR: Epidermal growth factor receptor
FasL: Fas ligand
α-GalCer: α-galactosylceramide
GVHD: Graft-versus-host disease
HEIC: Hapten-enhanced intratumoral chemotherapy
HPV: Human papillomavirus
HNSCC: Head and neck squamous cell carcinoma
HSCs: Hematopoietic stem cells
ICD: Immunogenic cell death
ICIs: Immune checkpoint inhibitors
iNKT: invariant natural killer T
IP3: Inositol trisphosphate
iPSCs: Induced pluripotent stem cells
iPSC-NK: Induced pluripotent stem cells (iPSC)-derived NK
ITAMs: Immune receptor tyrosine-based activation motifs
KIRs: Killer cell immunoglobulin-like receptors
KLRG1: Killer cell lectin-like receptor G1
NCRs: Natural cytotoxicity receptors
NK: Natural killer
NKG2D: Natural killer group 2, member D
MHC-I: Major histocompatibility complex class I
MDSCs: Myeloid-derived suppressor cells
MRD: Minimal residual disease
OPSCC: Oropharyngeal squamous cell carcinoma
PBNK: NK cells from peripheral blood
PD-1: Programmed cell death protein 1
γ-PGA: Poly-gamma-glutamic acid
PIP2: Phosphatidylinositol 4,5-bisphosphate
PI3K: Phosphatidylinositol 3-kinase
PKC: Protein kinase C
PLC-γ: Phospholipase C-γ
PRRs: Pattern recognition receptors
PVR: Poliovirus receptor
Rb: Retinoblastoma protein
RIG-I: Retinoic acid-inducible gene I
scRNA-Seq: Single-cell RNA sequencing
TAM: Tumor-associated macrophages
TGF-βR: TGF-β receptor
Tfh: T follicular helper cells
Tregs: Regulatory T cells
TIGIT: T cell immunoreceptor with Ig and ITIM domains
TIM-3: T-cell immunoglobulin and mucin-domain containing-3
TLR: Toll-like receptor
TME: Tumor microenvironment
TRAIL: TNF-related apoptosis-inducing ligand
trNK: Tissue-resident NK cell
UCB-NK: NK cells from umbilical cord blood
